# The *B. subtilis* translesion polymerase Pol Y1 is not strongly recruited to sites of replication upon different types of DNA damage

**DOI:** 10.1371/journal.pgen.1012246

**Published:** 2026-07-14

**Authors:** Sophia R. Martinez-Whitman, Chloe M. Santana, Alyssa P. Campbell, Denholm T. Feldman, Isaac E.Z. Jabaley, Luke G. O’Neal, McKayla E. Marrin, Elizabeth S. Thrall

**Affiliations:** Department of Chemistry and Biochemistry, Fordham University, Bronx, New York, United States of America; University of Wisconsin-Madison, UNITED STATES OF AMERICA

## Abstract

One challenge to DNA replication is the presence of unrepaired damage on the template strand, which can stall the replication machinery. This stall can be resolved by the translesion synthesis (TLS) pathway, in which specialized translesion polymerases are recruited to copy damaged DNA. Because TLS polymerases are error-prone, their activity is regulated at multiple levels to minimize unnecessary mutagenesis. Although the molecular mechanisms of bacterial TLS have been extensively studied in *Escherichia coli*, less is known about this pathway in other species. In *E. coli*, the TLS polymerase Pol IV is minimally enriched at replication forks in the absence of DNA damage but is strongly recruited upon replication stalling, enabling TLS while minimizing mutagenesis. However, we recently showed that the *Bacillus subtilis* TLS polymerase Pol Y1, the homolog of Pol IV, is moderately enriched near replication sites even during normal growth and is not further enriched upon treatment with the DNA damaging agent 4-nitroquinoline 1-oxide (4-NQO). It is unknown whether this behavior is unique to 4-NQO or general to other types of DNA damage. In this study, we investigate the effects of four different DNA damaging agents (ultraviolet light, methyl methanesulfonate, nitrofurazone, and mitomycin C) in *B. subtilis*. We first characterize the contributions of the two TLS polymerases, Pol Y1 and Pol Y2, to DNA damage survival and damage-induced mutagenesis after treatment with these agents. We then use single-molecule fluorescence microscopy to measure the localization and dynamics of individual Pol Y1 molecules in live *B. subtilis* cells. We find that Pol Y1 and Pol Y2 have differing effects on survival and mutagenesis, but that under no circumstances is Pol Y1 strongly recruited to sites of replication upon DNA damage. This study broadens our understanding of TLS in *B. subtilis*, indicating that there are notable differences in TLS mechanisms across bacteria.

## Author summary

Unrepaired DNA damage can stall the process of normal DNA replication, ultimately leading to cell death. One pathway for resolving this stall is translesion synthesis (TLS), in which specialized translesion polymerases copy the damaged DNA. Although it promotes cell survival, this pathway is mutagenic and therefore must be tightly regulated. TLS is conserved across all domains of life, but most studies of bacterial TLS are performed in *Escherichia coli*. Previous work in *E. coli* showed that TLS polymerases are only recruited to sites of DNA replication in the presence of DNA damage. In this study, we investigate TLS in the bacterium *Bacillus subtilis*. We first use microbiological assays to determine the contributions of the TLS polymerases Pol Y1 and Pol Y2 to tolerance of DNA damage and to damage-induced mutagenesis for four different types of DNA damage, finding that they have differing effects. We then use single-molecule fluorescence microscopy in live cells to determine whether Pol Y1 is recruited to replication sites upon damage. In contrast to *E. coli*, we find that Pol Y1 is not recruited upon DNA damage. These results provide new insight into TLS in *B. subtilis* and reveal that TLS mechanisms differ across bacterial species.

## Introduction

Unrepaired DNA damage can stall the replication machinery, a multi-protein complex known as the replisome, ultimately leading to cell death. Translesion synthesis (TLS) is a DNA damage tolerance pathway that promotes cell survival by alleviating this stall [1–3]. In this process, specialized translesion polymerases, generally members of the error-prone Y family, are recruited to copy damaged DNA, allowing replication to continue past blocking lesions. Although TLS promotes cell survival, TLS polymerases are generally low-fidelity, and therefore TLS is a mutagenic process. Thus, cellular regulation of TLS polymerases must strike a balance between minimizing their activity during normal unstressed conditions while also allowing them access to the DNA template when replication is stalled.

In bacteria, TLS has primarily been studied in the model gram-negative species *Escherichia coli*. In *E. coli*, it is clear that TLS polymerases are regulated at multiple levels to maintain genome stability while also allowing them access to stalled replication forks [2]. In particular, the TLS polymerase Pol IV is regulated transcriptionally via the SOS DNA damage response, which increases its cellular copy number approximately 10-fold after SOS induction. In addition to this transcriptional regulation, recent single-molecule imaging studies have also revealed a spatial component to Pol IV regulation. Even when Pol IV copy number is held constant, Pol IV is not strongly enriched at sites of replication during normal growth but is strongly enriched upon DNA damage or other replication perturbations, even for non-cognate DNA lesions that it cannot bypass [4–6]. These mechanisms ensure that Pol IV can gain access to the DNA template upon replication stalling, while limiting its access during unperturbed replication.

Although the molecular mechanisms of TLS have been elucidated in great detail in *E. coli*, much less is known about TLS in other bacterial species. The model gram-positive bacterium *Bacillus subtilis* is an attractive species to serve as an alternative model for bacterial TLS, given its evolutionary distance from *E. coli*. *B. subtilis* has two TLS polymerases, Pol Y1 and Pol Y2, which are the homologs of *E. coli* Pol IV and Pol V, respectively [7–9]. A few studies have started to reveal similarities and differences in the contributions of Pol Y1 and Pol Y2 to DNA damage survival and mutagenesis relative to their *E. coli* homologs. Like Pol IV, Pol Y1 promotes survival to the drug 4-nitroquinoline 1-oxide (4-NQO) [10,11]. However, unlike Pol IV, Pol Y1 promotes survival upon ultraviolet C (UV-C) treatment, whereas Pol Y2 deletion has no effect [10,12,13]. In contrast, *E. coli* Pol V mediates survival to UV damage [14,15]. Both Pol Y1 and Pol Y2 were shown to play a role in the tolerance of hexavalent chromium (Cr(VI))-mediated damage [16]. One study investigated DNA damage tolerance and mutagenesis in sporulating cells, rather than vegetatively growing cells, finding that both Pol Y1 and Pol Y2 had an effect on survival and mutagenesis for damaging agents like UV-C light, mitomycin C (MMC), and *tert*-butyl hydroperoxide [12].

In addition to differences in the functional roles of Pol Y1 and Pol Y2, it is becoming clear that there are differences in their cellular regulation. Unlike the *E. coli* TLS polymerases and Pol Y2, Pol Y1 is not a member of the SOS regulon [8,17]. Further, although Pol V is composed of two different subunits, the catalytic subunit UmuC and the accessory subunit UmuD, [2] Pol Y2 is currently thought to function as a single gene product [8]. There is also evidence that the spatial regulation of Pol Y1 differs from that of Pol IV. In a previous study, we found that Pol Y1 is moderately enriched at replication sites during normal growth, but that it is not further enriched upon 4-NQO treatment, [11] in contrast to Pol IV [4,6]. Similarly, we found no changes in Pol Y1 mobility upon 4-NQO treatment, [11] whereas DNA damage was shown to lead to increased Pol IV binding [4]. However, it is not known whether this lack of Pol Y1 recruitment is unique to 4-NQO or whether it is a general phenomenon.

In this study, we determine the contributions of Pol Y1 and Pol Y2 to DNA damage survival and mutagenesis in response to treatment with four different DNA damaging agents: UV light, methyl methanesulfonate (MMS), nitrofurazone (NFZ), and MMC. In matched experiments, we image single Pol Y1 molecules in live *B. subtilis* cells and measure their cellular localization and mobility. Although Pol Y1 and Pol Y2 deletions have differing effects on cell survival and mutagenesis upon different types of DNA damage, we find relatively modest changes in Pol Y1 mobility and in the enrichment of Pol Y1 at replication sites, even for cognate DNA damaging agents. These results indicate that the lack of Pol Y1 recruitment observed previously for 4-NQO is consistent for other types of DNA damage and reveal further differences in the mechanisms of TLS in *E. coli* and *B. subtilis*.

## Results

### Pol Y1 deletion has differing effects on cell survival, whereas Pol Y2 deletion has no effect

**Fig 1:**
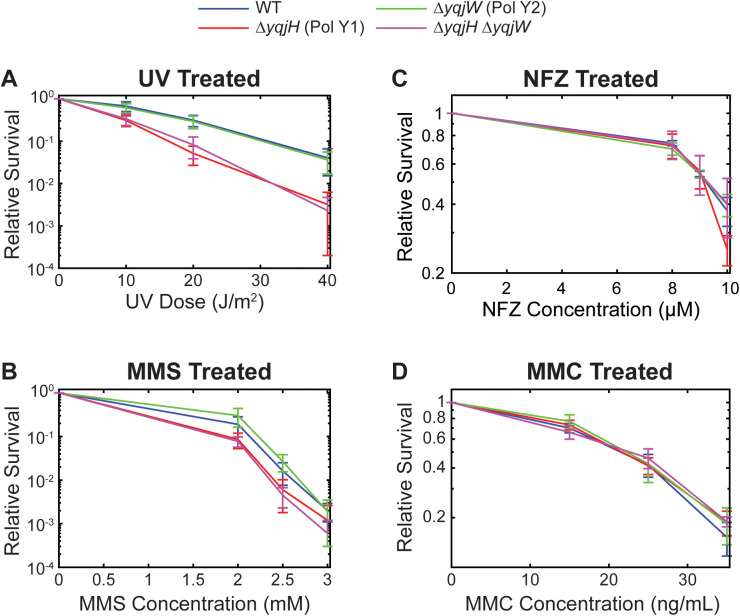
Survival of *B. subtilis* strains in the presence of different types of DNA damage. Relative survival rates for WT, ΔPol Y1 knockout, ΔPol Y2 knockout, and ΔPol Y1 ΔPol Y2 double knockout strains after treatment with different doses of **(A)** 254 nm UV light, **(B)** MMS, **(C)** NFZ, and **(D)** MMC. Error bars show standard deviation of at least three replicates. Note that all *y*-axes are on a log scale.

To survey the contributions of Pol Y1 and Pol Y2 to cell survival upon DNA damage, we decided to test treatment with four different DNA damaging agents that generate a range of different lesions. First, we tested the effect of UV irradiation, which generates highly blocking DNA lesions like cyclobutane pyrimidine dimers and 6–4 photoproducts [18]. In *E. coli*, Pol IV does not contribute to UV survival; instead, Pol V performs TLS past UV-induced lesions [14,15,19]. In *B. subtilis*, previous work by our lab [13] and others [7,10] has shown that only Pol Y1 promotes survival to UV damage, in contrast to *E. coli*. We assayed survival of cells to 0, 10, 20, and 40 J/m^2^ doses of 254 nm UV-C light (Fig 1A) and compared the WT strain to strains bearing knockouts of Pol Y1 (Δ*yqjH*) and Pol Y2 (Δ*yqjW*) individually or in combination (Δ*yqjH* Δ*yqjW*). Consistent with prior reports, [7,10] we found that deletion of Pol Y1 sensitized cells to UV (by roughly 10-fold at the highest dose; *p* < 0.05 at all doses), whereas deletion of Pol Y2 had no effect (*p* > 0.05 at all doses). The sensitivity of the double knockout alone was comparable to that of the single Pol Y1 knockout (*p* > 0.05 at all doses), indicating that Pol Y2 cannot substitute for Pol Y1 to bypass UV lesions.

Next, we tested the effect of treatment with the alkylating agent MMS, which generates various DNA lesions, including the replication-blocking *N*^3^-methyladenine [20,21]. In *E. coli*, Pol IV bypasses MMS lesions and promotes cell survival, whereas Pol V does not contribute to tolerance [22]. However, no prior work has investigated the effect of Pol Y1 and Pol Y2 on MMS survival or mutagenesis in *B. subtilis*. We assayed survival of cells deposited on LB agar plates containing MMS at concentrations of 0, 2, 2.5, and 3 mM (Fig 1B). As for UV treatment, we found that deletion of Pol Y1 sensitized cells to MMS, albeit more modestly (approximately two- to three-fold; *p* < 0.05 at 2 and 2.5 mM, *p* > 0.05 at 3 mM), whereas deletion of Pol Y2 had no effect (*p* > 0.05 at all concentrations). The sensitivity of the double knockout was comparable to that of Pol Y1 alone (*p* > 0.05 at all concentrations), again indicating that Pol Y2 cannot substitute for Pol Y1 to bypass MMS lesions.

The drug NFZ generates DNA strand breaks as well as small minor groove lesions, like *N*^2^ adducts of guanine, [23,24] which are efficiently bypassed by *E. coli* Pol IV, whereas Pol V does not promote survival [15,25]. Although NFZ is a well-studied cognate damaging agent for Pol IV, no prior work has investigated the effect of Pol Y1 and Pol Y2 on NFZ survival in *B. subtilis*. Therefore, we assayed survival of cells plated on LB agar plates containing NFZ at concentrations of 0, 8, 9, and 10 µM (Fig 1C). The survival rate was relatively high (~ 35 – 40%) at the highest concentration and dropped precipitously above 10 µM. However, we were unable to obtain consistent assay results at higher NFZ concentrations. Within this limited concentration range, there was no obvious effect of Pol Y1 or Pol Y2 deletion, either individually or in combination (*p* > 0.05 at all concentrations). Thus, in contrast to the behavior of its *E. coli* homolog, Pol Y1 does not appear to confer survival to NFZ damage, at least in the regime of relatively high survival rates.

Finally, we tested the effect of Pol Y1 and Pol Y2 deletion on survival upon treatment with the drug MMC. MMC is an alkylating agent that generates a wide range of different types of lesions, including both interstrand crosslinks (ICLs) and intrastrand crosslinks as well as *N*^2^ and *N*^7^ monoadducts of guanine [26–29]. To our knowledge, there are no reports on the roles of *E. coli* Pol IV and Pol V in tolerance of MMC-induced damage. In *B. subtilis*, no studies have tested the effect of Pol Y1 and Pol Y2 on survival or mutagenesis in vegetative cells, although both contribute to survival in sporulating cells [12]. We assayed survival of cells to 0, 15, 25, and 35 ng/mL concentrations of MMC in solid media (Fig 1D). At the highest of these concentrations, the relative survival of the WT strain was still relatively high, at approximately 0.15. We also tested a higher concentration of 45 ng/mL; however, at this concentration, survival dropped precipitously and the colonies became translucent and challenging to count. Thus, we did not use concentrations greater than 35 ng/mL. Over the range of concentrations, deletion of Pol Y1, Pol Y2, or both had no effect on MMC survival (*p* > 0.05 at all concentrations), in contrast to a previous report in sporulating cells [12]. These results suggest that Pol Y1 and Pol Y2 may play different roles during sporulation and vegetative growth.

### Pol Y1 and Pol Y2 both contribute to damage-induced mutagenesis under different conditions

**Fig 2:**
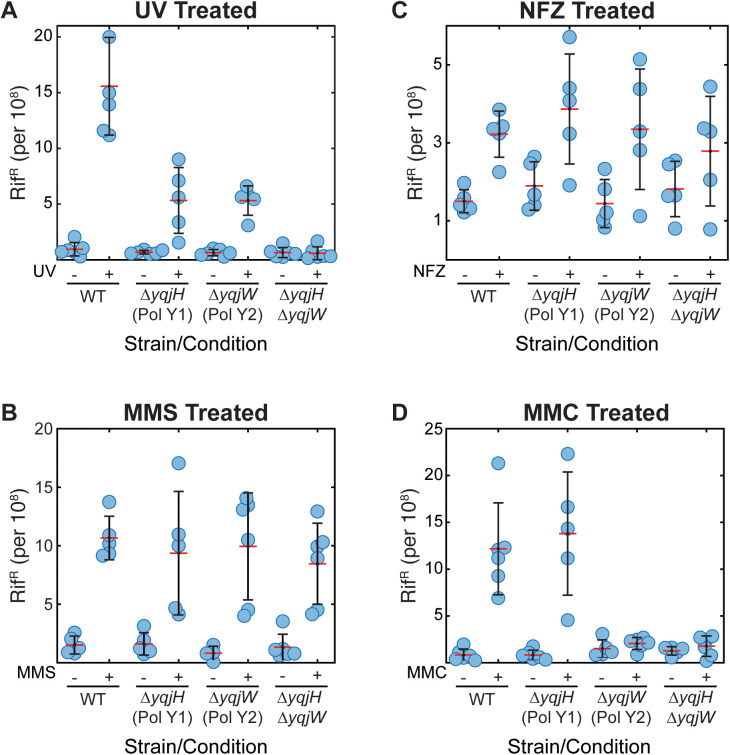
Relative mutation frequencies for *B. subtilis* strains in the presence of different types of DNA damage. Proportion of rifampicin resistant (Rif^R^) cells for WT, ΔPol Y1 knockout, ΔPol Y2 knockout, and ΔPol Y1 ΔPol Y2 double knockout strains in untreated cells and after treatment with **(A)** 40 J/m^2^ 254 nm UV light, **(B)** 10 mM MMS, **(C)** 100 µM NFZ, and **(D)** 200 ng/mL MMC. For each dataset, the individual replicates are shown as circles, the red line represents the mean value, and the error bars represent the standard deviation of at least five replicates.

In addition to surveying the roles of Pol Y1 and Pol Y2 in damage tolerance, we also characterized their effect on damage-induced mutagenesis upon treatment with the same set of damaging agents. We first explored UV-induced mutagenesis, which in *E. coli* is mediated by Pol V [19,30]. Prior results on UV-induced mutagenesis in *B. subtilis* were not in agreement. One study found that both Pol Y1 and Pol Y2 contribute, with Pol Y1 having the greater impact, [7] whereas another study reported that Pol Y2 alone was responsible for UV-induced mutagenesis [8]. We assayed mutagenesis in untreated cells and cells treated with a 40 J/m^2^ dose of 254 nm UV-C light, matching prior literature reports, by quantifying the proportion of cells that were resistant to the drug rifampicin (Rif^R^) (Fig 2A and Table A in S1 Text). Consistent with these prior reports, the Rif^R^ frequency for the wild-type (WT) strain was approximately one in 10^8^ in untreated cells and increased by 15-fold in UV-treated cells (*p* < 0.05). Deletion of either Pol Y1 or Pol Y2 alone led to a 3-fold reduction in UV-mutagenesis relative to WT (*p* < 0.05), broadly consistent with the results of Sung, et al. [7] Notably, deletion of both Pol Y1 and Pol Y2 eliminated any UV-induced mutagenesis (*p* < 0.05), indicating that Pol Y1 and Pol Y2 act in separate pathways to generate mutations upon UV treatment.

Next, we measured mutagenesis induced by MMS treatment. In *E. coli*, there is a modest reduction in mutagenesis in the absence of Pol V, but a substantial (approximately order of magnitude) increase in mutagenesis upon Pol IV deletion, indicating that Pol V performs highly error-prone synthesis in the absence of Pol IV [22]. We assayed mutagenesis in cells treated with 10 mM MMS for 1 h for the same set of four strains (Fig 2B and Table A in S1 Text). We found that the Rif^R^ frequency for all strains increased by approximately 5 – 10-fold upon MMS treatment, to approximately one in 10^7^ (*p* < 0.05). However, although cells lacking Pol Y1 were modestly sensitized to MMS treatment in survival assays, we found no effect of either Pol Y1 or Pol Y2 deletion on MMS-induced mutagenesis (*p* > 0.05). Likewise, the double Pol Y1 and Pol Y2 knockout had an MMS-induced mutation frequency that was statistically indistinguishable from WT (*p* > 0.05). Thus, although Pol Y1 acts to confer tolerance to MMS-induced DNA damage, it does not contribute to mutagenesis.

In *E. coli*, NFZ was reported to induce mutagenesis more weakly than MMS [31]. Surprisingly, although Pol IV promotes survival to NFZ treatment, it has no impact on NFZ-induced mutagenesis [32]. To our knowledge, no prior work has studied the effect of Pol Y1 and Pol Y2 on NFZ-induced mutagenesis in *B. subtilis*. We quantified mutagenesis in cells treated with 100 µM NFZ for 1 h (Fig 2C and Table A in S1 Text). For the WT strain, there was a small (approximately 2-fold) but statistically significant (*p* < 0.05) increase in the Rif^R^ frequency upon NFZ treatment; the same was true for the Pol Y1 knockout strain. Although similar increases were also observed for the Pol Y2 knockout strain and the double knockout, they did not reach statistical significance at the *p* < 0.05 level. However, the NFZ-induced Rif^R^ frequency was statistically indistinguishable for all three knockout strains relative to WT (*p* > 0.05), indicating no effect of Pol Y1 or Pol Y2 on NFZ-induced mutagenesis; these results are consistent with the survival assay results showing no effect of Pol Y1 and/or Pol Y2 deletion on tolerance of NFZ-induced DNA damage.

Finally, we assayed mutagenesis upon MMC treatment. As for MMC survival, we are unaware of any studies in *E. coli* on the roles of Pol IV and Pol V in MMC-induced mutagenesis. In *B. subtilis*, both Pol Y1 and Pol Y2 contribute to MMC-induced mutagenesis in sporulating cells, [12] but no studies have investigated their effect in vegetative cells. We assayed mutagenesis in cells treated with 200 ng/mL MMC for 1 h (Fig 2D and Table A in S1 Text), a condition used previously in other mutagenesis experiments [33] as well as microscopy experiments [34] in *B. subtilis*. For the WT strain, MMC treatment increased the mutation frequency by approximately 15-fold (*p* < 0.05); the same was true for the Pol Y1 knockout strain, which was statistically indistinguishable from WT. However, there was a complete loss of MMC-induced mutagenesis for the Pol Y2 deletion, either on its own or in the double knockout strain (*p* > 0.05 for untreated vs. MMC-treated and *p* < 0.05 for MMC-treated relative to WT). Thus, although Pol Y2 does not contribute to survival upon MMC treatment, Pol Y2 is entirely responsible for MMC-induced mutagenesis.

### DNA damage induces the SOS response

Pol Y2 is present at very low copy number in the absence of DNA damage, but it is upregulated upon induction of the SOS response [7,8,17]. Therefore, to test whether the treatments used in these mutagenesis assays induce SOS, and thus Pol Y2 expression, we imaged cells bearing a previously characterized SOS reporter construct. In this transcriptional P_*yneA*_-*gfp* reporter, GFP expression is under the control of the promoter of *yneA*, an SOS-regulated gene that inhibits cell division upon DNA damage [35]. As expected, we observed low basal fluorescence in normally growing cells, but a strong increase upon DNA damage. These results are consistent with prior reports that treatment with UV, MMC, and the alkylating agent ethyl methanesulfonate (EMS) all induce SOS in *B. subtilis* [36] and that NFZ and MMS treatment induce SOS in *E. coli* [31]. To quantify the degree of SOS induction, we calculated the normalized cellular fluorescence by measuring the total fluorescence intensity for each cell and dividing by the cell area (S1 Fig). The mean normalized cellular fluorescence intensity increased relative to the untreated condition by a factor of 3.4-fold upon UV treatment, 1.6-fold upon MMS treatment, 2.1-fold upon NFZ treatment, and 3.3-fold upon MMC treatment (*p* < 0.05 in all cases; fold-changes in median intensities were almost identical). Thus, all four treatment conditions induce SOS, with UV and MMC leading to greater induction than NFZ and MMS. Taken together, these results suggest that the lack of contribution of Pol Y2 to survival for all four damaging agents and to mutagenesis for MMS and NFZ does not simply reflect a lack of expression; for example, 40 J/m^2^ UV treatment strongly induces SOS, yet Pol Y2 has no impact on cell survival under this condition (Fig 1A).

### DNA damage leads to coupled changes in the cellular localization of Pol Y1 and the replisome

**Fig 3:**
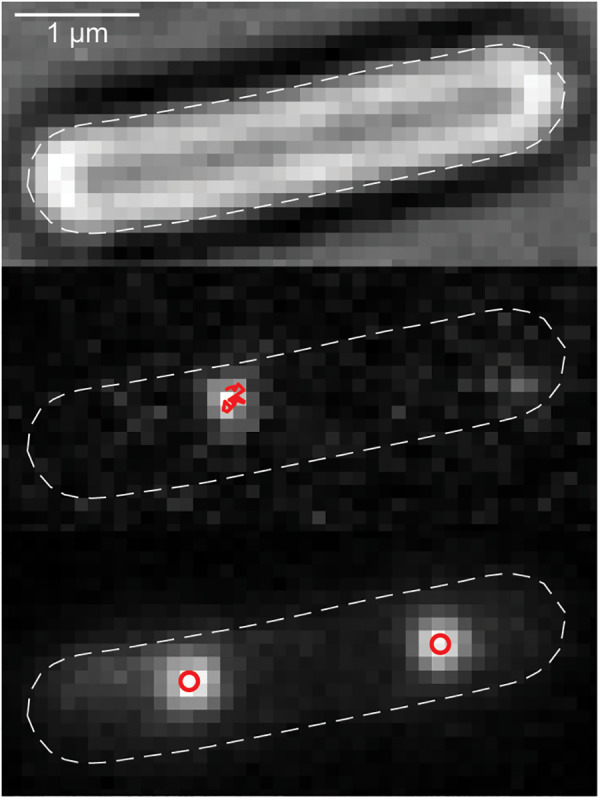
Representative micrographs of Pol Y1 and DnaX. (Top) Transmitted white light micrograph of *B. subtilis* cell with overlaid cell outline and 1 µm scale bar. (Middle) Fluorescence micrograph of single Pol Y1-Halo-JFX_554_ molecule with overlaid trajectory. (Bottom) Fluorescence micrograph of DnaX-mYPet foci with overlaid centroids.

Having characterized the effect of Pol Y1 and Pol Y2 deletion on cell survival and mutagenesis upon treatment with different types of DNA damage, we next explored the effect of these treatments on the localization of the replisome and Pol Y1 in live cells (Fig 3, top panel). As a marker for sites of replication, we used the core replisome component DnaX, a subunit of the clamp-loader complex, fused to the YFP variant mYPet [37]. Prior work by us [11,13] and others [38–41] has shown that DnaX forms distinct foci at sites of replication (Fig 3, bottom panel). To image single Pol Y1 molecules, we used a C-terminal fusion to the HaloTag, labeled with the Janelia Fluor X 554 (JFX_554_) dye (Fig 3, middle panel); [42] we validated this imaging strategy previously, showing that the Pol Y1 fusion retains full functionality and that the JFX_554_ labeling procedure has no detectable effects on cell growth or morphology [11]. As additional confirmation that the HaloTag does not affect Pol Y1 function, we assayed the survival of fusion strains upon UV treatment. We found that the Pol Y1-Halo fusion had no effect on cell survival upon UV treatment relative to WT, either in the presence or absence of the DnaX-mYPet fusion (S2 Fig), validating its use in microscopy.

**Fig 4:**
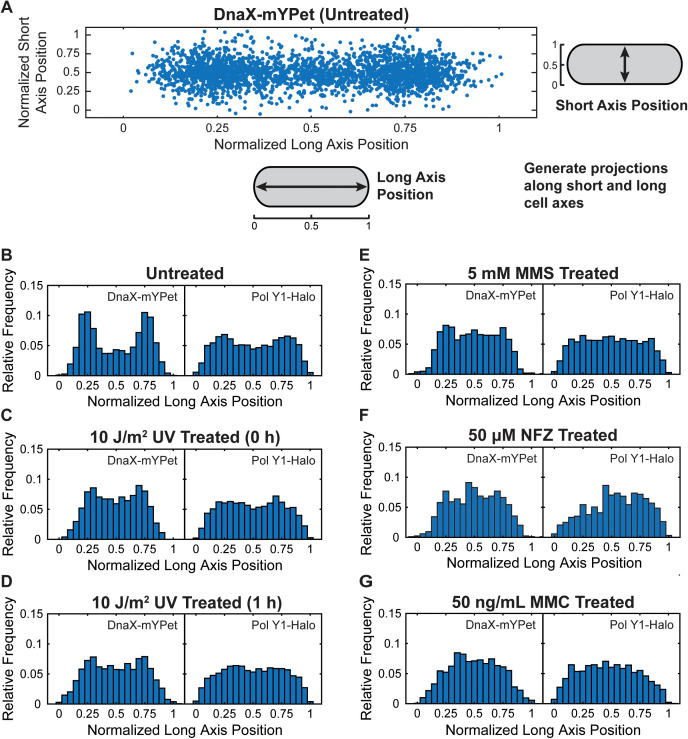
Average cellular localization of DnaX-mYPet and Pol Y1-Halo in the presence of different types of DNA damage. **(A)** Scatter plot of normalized positions of DnaX foci in untreated cells and cartoon of long and short cell axis projections. Long axis projections of DnaX foci (left) and all detected Pol Y1 trajectories (right) in **(B)** untreated cells and after treatment with **(C)** 10 J/m^2^ 254 nm UV light (*t* = 0 h), **(D)** 10 J/m^2^ 254 nm UV light (*t* = 1 h), **(E)** 5 mM MMS, **(F)** 50 µM NFZ, and **(G)** 50 ng/mL MMC. The corresponding short axis projections are shown in S3 Fig.

As an initial step, we measured the cellular localization of Pol Y1 and DnaX. Consistent with our prior work, we found that untreated cells growing in minimal S7_50_-sorbitol media typically contained one or two DnaX foci (mean ± S.E.M.: 1.76 ± 0.02; Table B in S1 Text). We calculated the average cellular position of DnaX foci by generating a normalized set of coordinates along the long and short cell axes for each cell (Fig 4A) and found that foci were generally localized at the quarter and three-quarter positions along the long-cell axis (Fig 4B) and at mid-cell along the short-cell axis (S3A Fig), consistent with expectations for normally growing cells. Also consistent with our prior work, we found that Pol Y1 had a similar average localization pattern to DnaX, albeit with a somewhat broader distribution (Fig 4B) [11].

Next, we asked how the location of replication sites and Pol Y1 were altered by UV treatment. We tested the effect of treating cells with two different UV doses, 10 and 20 J/m^2^, imaging cells both immediately after exposure and after 1 h of further growth to detect delayed effects of UV treatment. These doses are in a range used in prior imaging studies in *E. coli* [6,43]. However, to determine the effect of these treatments on cell survival in *B. subtilis*, we plated cells before and after UV exposure and quantified the fold-change in the number of CFUs/mL. As reported previously, the number of CFUs/mL doubled (mean ± std.: 2.1 ± 0.4; Table C in S1 Text) under these growth conditions in the absence of any perturbations [11]. We found that the 10 J/m^2^ UV dose led to a moderate reduction in cell viability immediately after treatment (0.72 ± 0.08), whereas the higher 20 J/m^2^ dose led to a greater reduction (0.4 ± 0.1). However, the number of CFUs/mL approximately doubled after 1 h for both treatments, consistent with the increase observed in untreated cells, indicating a recovery of growth. We note that these UV doses are lower than the 40 J/m^2^ dose used in our mutagenesis assays. Although this higher dose was selected to match prior studies of Pol Y1 and Pol Y2 mutagenesis, we chose not to use it in imaging experiments due to the significant loss of viability observed at the lower 20 J/m^2^ dose, raising the concern that imaging results might not be physiologically relevant if a substantial majority of cells were non-viable. We quantified the cell morphology and the average number of DnaX foci per cell after these two UV treatments, finding that there were moderate increases (approximately 20%) in cell length and in the number of DnaX foci for both doses at the 1 h timepoint (Table B in S1 Text). For the 10 J/m^2^ dose, there were minor changes in DnaX and Pol Y1 localization at both timepoints, with long-axis localization still peaking at the quarter and three-quarter cell positions, albeit with slightly more mid-cell localization (Fig 4C and 4D). However, for the 20 J/m^2^ dose, both DnaX and Pol Y1 localization shifted more strongly to mid-cell along the long cell axis (S4A and S4B Fig), retaining mid-cell localization along the short-cell axis (S3D and S3E Fig); this shift is consistent with perturbations to replication and was previously observed in cells treated with 4-NQO [11].

Next, we tested the effect of treating cells with three different concentrations of MMS (5, 10, and 15 mM) for 1 h in liquid culture; these concentrations are in a range previously used in imaging studies in *E. coli* [44] and *B. subtilis* [45]. The lowest concentration (5 mM) slowed growth but did not lead to a loss of cell viability (1.4 ± 0.3; Table C in S1 Text), the intermediate concentration (10 mM) led to a modest reduction in viability (0.9 ± 0.1), and the highest concentration (15 mM) led to a substantial reduction (0.30 ± 0.03). There were minimal changes in cell length (< 10% reduction) for all three concentrations (Table B in S1 Text). Although there was no change in the number of DnaX foci per cell at the lowest concentration, there were moderate (15 – 20%) reductions at the higher two concentrations. All three treatments led to a coupled shift in both DnaX and Pol Y1 localization toward mid-cell along the long-cell axis, but with the shift becoming more pronounced as MMS concentration increased (Fig 4E and S4C and S4D Fig).

For NFZ, we found that treatment with a wide range of concentrations from 50 to 250 µM slowed growth but did not lead to substantial loss of viability (Table C in S1 Text); as expected, treatment with the dimethylformamide (DMF) solvent alone had no effect. Thus, we decided to test exposure to two concentrations, 50 and 100 µM, for 1 h in liquid culture, both of which stalled growth without substantial cell killing (1.1 ± 0.3 and 1.0 ± 0.1, respectively); these conditions are similar or identical to NFZ treatments used in prior imaging studies of *E. coli* Pol IV [4,46]. The higher concentration led to a modest (approximately 15% reduction) in cell length and somewhat larger (approximately 15 – 25%) reductions in the number of DnaX foci per cell (Table B in S1 Text). For the 50 µM NFZ concentration, there was a shift in both DnaX and Pol Y1 localization toward mid-cell along the long cell axis (Fig 4F); for the 100 µM concentration, there was a similar shift for DnaX, although less so for Pol Y1 (S4E Fig).

Finally, we tested the effect of treating cells with three different concentrations of MMC (50, 100, and 200 ng/mL) for 1 h in liquid culture. The lower two concentrations were used in prior imaging studies of *B. subtilis*, [47,48] whereas the higher concentration was used in prior mutagenesis [33] and imaging experiments [34]. These concentrations lead to increasing loss of viability (0.8 ± 0.2, 0.5 ± 0.1, and 0.18 ± 0.07, respectively; Table C in S1 Text). We confirmed that treatment with the dimethyl sulfoxide (DMSO) solvent alone did not affect growth. Given the significant reduction in viability at the highest concentration, we decided to use only the lower two concentrations in imaging experiments, even though we used the higher concentration in mutagenesis assays to match prior studies in *B. subtilis*. As for UV treatment, we cannot exclude the possibility that this modest difference in MMC dose leads to qualitative differences in Pol Y1 behavior, although we believe that it is unlikely. Both MMC treatments used in imaging experiments led to a moderate (~ 20% increase) in cell length, but no statistically significant changes in the number of DnaX foci per cell (Table B in S1 Text). The DnaX and Pol Y1 average localization was similar for both treatments (Fig 4G and S4F Fig), with a shift in localization toward mid-cell along the long cell axis, indicative of perturbed replication.

### DNA damage does not induce substantial changes in Pol Y1 colocalization with sites of replication

As described in the previous section, measurements of average cellular localization showed concomitant shifts in the position of replication sites and Pol Y1 upon increasing levels of DNA damage. However, these qualitative comparisons do not allow us to quantify changes in Pol Y1 enrichment at replication sites. Thus, we used the radial distribution function approach to measure the degree of Pol Y1-DnaX colocalization. In brief, the radial distribution function *g*(*r*) reports on the colocalization of two proteins in the cell relative to the colocalization that would be observed by chance due to confinement. The *g*(*r*) value at a given separation distance, or radius, is the fold-enrichment relative to chance of Pol Y1 at that distance from the nearest DnaX focus. Thus, a *g*(*r*) value of 1 indicates no enrichment between the two proteins relative to chance, whereas *g*(*r*) > 1 at short separation distances indicates colocalization [4,49]. Because this analysis normalizes the experimental distribution of Pol Y1-DnaX distances by a random distribution calculated for each cell in the dataset, it is unaffected by changes in cell morphology or in the number or localization of DnaX foci.

**Fig 5:**
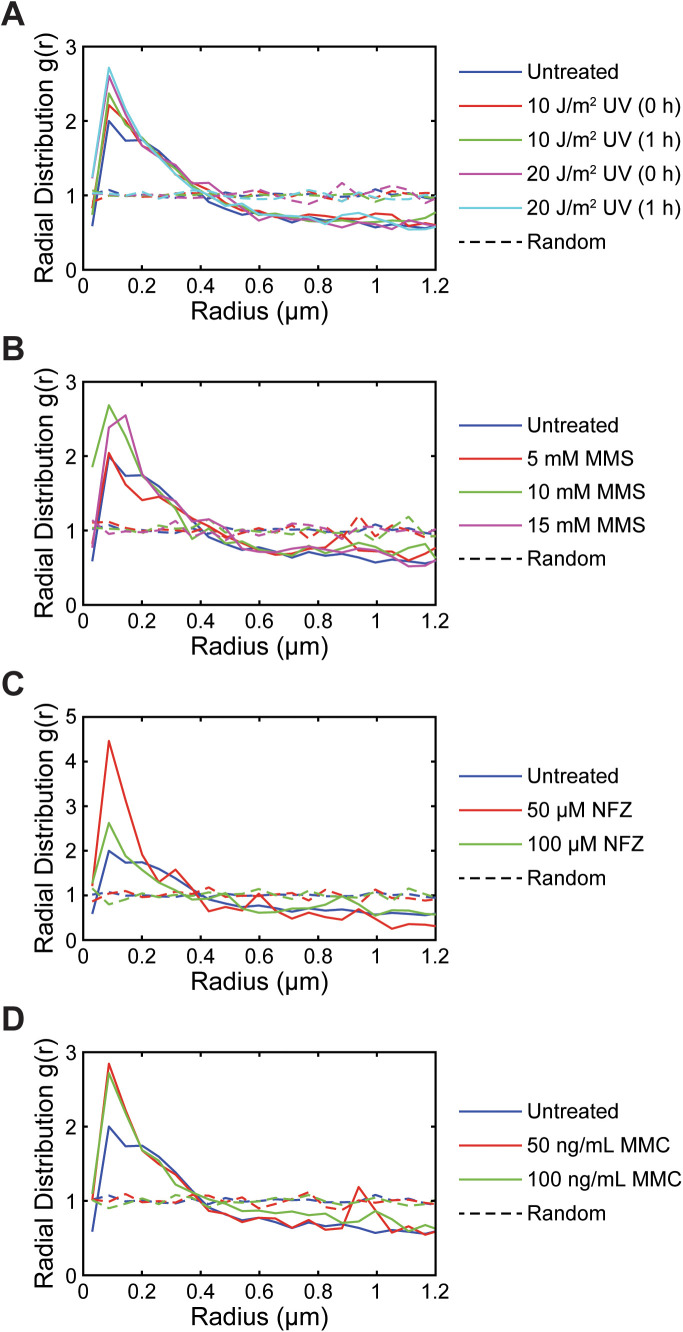
Radial distribution function *g*(*r*) analysis of Pol Y1-Halo and DnaX-mYPet colocalization in the presence of different types of DNA damage. Pol Y1-DnaX *g*(*r*) for all detected trajectories in untreated cells and **(A)** after treatment with 10 and 20 J/m^2^ 254 nm UV light (*t* = 0 and 1 h), **(B)** 5, 10, and 15 mM MMS, **(C)** 50 and 100 µM NFZ, and **(D)** 50 and 100 ng/mL MMC. Random *g*(*r*) curves are shown as dashed lines. Values of *g*(*r*) > 1 indicates colocalization, whereas *g*(*r*) = 1 indicates no colocalization. Note different *y*-axis scale in panel **(C)**.

In previous work, we measured the radial distribution function for Pol Y1 and DnaX in untreated cells and found *g*(*r*) ≈ 3 at short distances, indicative of a moderate level of colocalization between Pol Y1 and sites of replication; [11] we repeated these measurements in untreated cells and found *g*(*r*) ≈ 2.0 ± 0.07 (mean ± std. dev. of 100 calculated *g*(*r*) curves) (Fig 5A and Table D in S1 Text), in reasonable agreement with our prior report. For *E. coli* Pol IV, diverse types of DNA damage, and even damage-independent replication perturbation by the drug hydroxyurea, were shown to produce large increases, typically around 4 – 5-fold, in Pol IV colocalization with replication sites, even for non-cognate lesions that Pol IV cannot bypass [4,6]. However, we previously found that there was minimal change in Pol Y1-DnaX colocalization upon treatment with the drug 4-NQO [11].

To test whether this lack of damage-induced enrichment is a general phenomenon, we measured the Pol Y1-DnaX radial distribution function for all the different DNA damage treatments described previously. For UV treatment, both 10 and 20 J/m^2^ doses produced only modest increases in Pol Y1-DnaX colocalization at both timepoints; the increases for 20 J/m^2^ were marginally higher, with a maximum *g*(*r*) ≈ 2.7 ± 0.1 (Fig 5A). For MMS treatment, the *g*(*r*) curve for the lowest concentration (5 mM) was very similar to the untreated curve, whereas the 10 and 15 mM concentrations led to a slight increase in colocalization (maximum *g*(*r*) ≈ 2.7 ± 0.3) (Fig 5B). Results for NFZ were more mixed; there was a marked increase in colocalization (maximum *g*(*r*) ≈ 4.5 ± 0.8), but only for the lower concentration (50 µM), whereas the *g*(*r*) curve for the higher concentration (100 µM) was very similar to the untreated curve (Fig 5C). Finally, both MMC treatments led to a similar modest increase in Pol Y1-DnaX colocalization (maximum *g*(*r*) ≈ 2.8 ± 0.2) (Fig 5D). Taken together, these results show that there is no substantial increase in Pol Y1 enrichment at replication sites upon DNA damage, in contrast to prior findings for *E. coli* Pol IV. Further, they reveal no obvious differences in Pol Y1 enrichment between cognate (UV, MMS) and non-cognate (NFZ, MMC) DNA damaging agents.

### DNA damage has differing effects on Pol Y1 mobility in the cell

In previous studies of protein mobility in the cell, DNA polymerases and other DNA-binding proteins have been found to exist in two broad populations: a static population, immobilized via interactions with DNA or with other DNA-bound proteins, and a mobile population, diffusing throughout the cell [4,50]. Although not all static polymerase molecules are actively performing DNA synthesis, it is expected that molecules synthesizing DNA are found in this fraction. Previously, we characterized the static and mobile populations of Pol Y1 and found that static Pol Y1 molecules are enriched near sites of replication in the cell during normal growth [11]. In *E. coli*, DNA damage has been shown to increase the static population of Pol IV [4] and of DNA repair proteins like Pol I and ligase; [50] these static molecules are presumably binding at or near DNA lesions.

**Fig 6:**
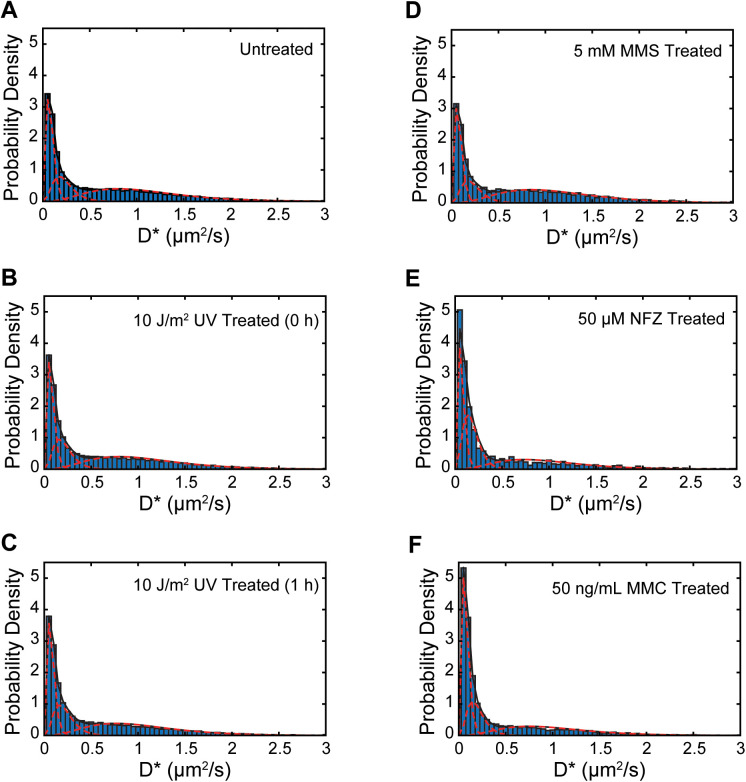
Apparent diffusion coefficient (D^*^) distributions and corresponding three-population fits for Pol Y1-Halo in the presence of different types of DNA damage. Pol Y1 D^*^ distributions for all detected trajectories in **(A)** untreated cells and after treatment with **(B)** 10 J/m^2^ 254 nm UV light (*t* = 0 h), **(C)** 10 J/m^2^ 254 nm UV light (*t* = 1 h), **(D)** 5 mM MMS, **(E)** 50 µM NFZ, and **(F)** 50 ng/mL MMC. Individual populations are shown as red dashed lines and overall fits are shown as solid black lines.

Before investigating the effect of DNA damage on Pol Y1 mobility, we first characterized Pol Y1 mobility in untreated cells. Following our previous approach, we calculated the apparent diffusion coefficient (D*) from the mean squared displacement of each trajectory (Fig 6A and Table E in S1 Text). We then fit the resulting D* distribution to a 3-population model; as we found previously, [11] and as others have seen for other DNA-binding proteins, [51] a 2-population model with a single static and mobile population does not adequately fit the distribution. The resulting fit yields a static DNA-bound population with low mobility (D* ≈ 0.08 µm^2^/s), a highly mobile population (D* ≈ 1.0 µm^2^/s), and an intermediate population (D* ≈ 0.2 µm^2^/s); this population may represent molecules interacting transiently with DNA. Consistent with our prior study, we found that static molecules represented ~ 30% of the population (32 ± 3% vs. 28 ± 3% previously) whereas mobile molecules represented almost half of the population (48 ± 3% vs. 47 ± 3% previously) [11].

Next, we asked whether treatment with DNA damaging agents altered the mobility of Pol Y1 molecules in the cell. Although there were changes in the relative populations of Pol Y1 upon DNA damage, most of these changes were relatively small (Fig 6B – 6F, S5A – S5F Fig and Table E in S1 Text). With only a few exceptions, the 95% fit confidence intervals for each population in damaged cells overlapped the corresponding intervals for each population in undamaged cells, suggesting that these small changes are not statistically significant. In particular, changes for both UV treatments were minimal, with the only statistically significant change being an approximately 20% decrease in the mobile population at the *t* = 1 h timepoint for the 20 J/m^2^ dose. MMS treatment likewise had only minor effects on Pol Y1 mobility, with only a small (~ 10%) increase in the mobile population for the 5 mM concentration appearing distinguishable from the untreated condition. NFZ treatment produced larger changes in Pol Y1 mobility than either UV or MMS, but only a decrease of approximately 30% in the mobile population for the 50 µM concentration appeared significant. MMC led to the largest changes in Pol Y1 diffusion, but without an obvious dose-dependent effect. The lower concentration (50 ng/mL) produced a larger increase in the static population (~ 40%), paired with a corresponding decrease in the mobile population (~ 30%), both of which were distinguishable from the untreated condition. The higher concentration (100 ng/mL) led to a similar decrease in the mobile population, which did reach statistical significance, but with a smaller (~ 10%) increase in the static population, which did not. Thus, DNA damage led to relatively modest changes in Pol Y1 mobility in the cell, with many of the measured changes not proving statistically significant.

### DNA damage has minimal effects on Pol Y1 binding lifetime

Although diffusion analysis did not reveal substantial changes in Pol Y1 mobility upon DNA damage, we also characterized the Pol Y1 binding lifetime under the different treatment conditions as an alternative measurement of its dynamics. In brief, we used a longer 250 ms integration time to visualize statically bound Pol Y1 molecules selectively and measured the duration of each trajectory. Because either Pol Y1 dissociation or JFX_554_ photobleaching could cause a trajectory to end, we corrected this apparent binding lifetime (*τ*_app_) by the dye photobleaching lifetime measured in fixed cells. We then fit the resulting distribution of corrected binding lifetimes to a single-exponential decay to determine the mean binding lifetime (*τ*_bound_) (S6 Fig and Table F in S1 Text). Consistent with our prior study, we found that *τ*_bound_ ≈ 2 s in untreated cells (1.95 ± 0.04 s vs. 1.9 ± 0.4 s previously) [11]. Previously, we showed that 4-NQO treatment had a minimal impact on the Pol Y1 binding lifetime. Likewise, none of the treatment conditions tested in this study led to a greater than approximately two-fold change in *τ*_bound_, and the average change was smaller (Table F in S1 Text). The 95% fit confidence intervals on *τ*_bound_ for all treatments overlapped with those in untreated cells, with one exception (10 J/m^2^ UV treatment, *t* = 0 h time point) indicating that the measured differences in binding lifetime may not be statistically significant. Thus, the binding lifetime analysis is consistent with the diffusion analysis in revealing minimal changes in Pol Y1 mobility upon DNA damage.

## Discussion

*E. coli* has served as the model system for bacterial TLS since the identification of translesion polymerases a quarter century ago, [1,2] yet it remains poorly understood how widely the mechanisms elucidated in *E. coli* hold in other species. In a previous study, we investigated the subcellular localization and dynamics of the *B. subtilis* TLS polymerase Pol Y1, the homolog of *E. coli* Pol IV, both during normal growth and after treatment with the cognate DNA damaging agent 4-NQO [11]. We also characterized the effect of deleting Pol Y1 or Pol Y2, the *E. coli* Pol V homolog, on 4-NQO survival and damage-induced mutagenesis, finding that Pol Y1 contributed to survival whereas neither polymerase had a significant effect on mutagenesis. In this study, we have expanded our investigation to include a range of different DNA damaging agents (UV, MMS, NFZ, and MMC) with the goal of broadening our understanding of TLS mechanisms in *B. subtilis*.

Because there is little or no literature data on treatment with these drugs in *B. subtilis*, we first determined the effect of Pol Y1 and Pol Y2 deletion, individually or in combination, on cell survival and mutagenesis after treatment with different doses of these four damaging agents. These experiments revealed several contrasts with *E. coli*, summarized in Table 1. Notably, Pol Y1 does not promote survival to NFZ treatment, which is a cognate damaging agent for its *E. coli* homolog Pol IV, yet it does promote survival to UV light, which in *E. coli* is mediated by Pol V. These results also demonstrate that Pol Y1 and Pol Y2 sometimes have differing effects on survival and mutagenesis upon treatment with the same drug; for example, Pol Y2 is responsible for MMC-induced mutagenesis but has no effect on MMC survival. Although it is surprising that a TLS polymerase could contribute to mutagenesis without affecting survival, there is at least one precedent in *E. coli*, in which Pol V deletion has no impact on MMS survival but does reduce mutagenesis [22]. Notably, we found no effect of either Pol Y1 or Pol Y2 deletion on MMC survival, in contrast to a prior report for sporulating cells, in which both contributed to survival, [12] suggesting that TLS mechanisms may differ during vegetative growth and sporulation in *B. subtilis*.

**Table 1:** Summary of the contributions of *E. coli* Pol IV and Pol V and *B. subtilis* Pol Y1 and Pol Y2 to DNA damage survival and mutagenesis for the damaging agents tested in this study. Key: some impact (+), no impact (−), no prior studies identified (?). (Prior studies in sporulating *B. subtilis* cells are excluded.).

Damaging Agent		*E. coli*			*B. subtilis*		
		Pol IV	Pol V	Refs.	Pol Y1	Pol Y2	Refs.
**UV**	Survival	−	+	(14,15,19)	+	−	(7,10,13)
	Mutagenesis	−	+	(19,30)	+	+	(7,8)
**MMS**	Survival	+	−	(22)	+	−	N/A
	Mutagenesis	−	+	(22)	−	−	N/A
**NFZ**	Survival	+	−	(15,25)	−	−	N/A
	Mutagenesis	−	?	(32)	−	−	N/A
**MMC**	Survival	?	?	N/A	−	−	N/A
	Mutagenesis	?	?	N/A	−	+	N/A

Our findings raise questions about the role of TLS in DNA damage tolerance in *B. subtilis*. Although results can be challenging to compare across different assay designs, deletion of Pol IV or Pol V in *E. coli* reduces survival for DNA damage induced by 4-NQO, [52] UV, [14] MMS, [22,25] and NFZ [4,15,32] by approximately 100 – 1,000-fold under treatment conditions that lead to minimal or moderate killing of WT cells. In contrast, for the same types of DNA damage, we observed reductions in survival of 10 – 50-fold at most for Pol Y1 knockouts in *B. subtilis*, and no effect of Pol Y2 deletion on survival. One possible explanation for this discrepancy is that other pathways supplement TLS by Y-family polymerases in *B. subtilis*, explaining the more modest effect of Pol Y1 and Pol Y2 deletion on cell survival. In particular, prior research has suggested that the replicative polymerase DnaE may have a role in DNA damage tolerance. Unlike most replicative polymerases, DnaE is a member of the SOS regulon and is modestly upregulated upon DNA damage; [17,53] it is also able to bypass certain types of DNA damage [53,54]. If DnaE is capable of acting as a TLS polymerase in addition to its role in replication, it may provide redundancy in damage tolerance. Although more speculative, it is also possible that Pol I plays a role in TLS. A previous study found that Pol I contributes to mutagenesis mediated by Pol Y1 and Pol Y2 under different conditions [55]. Further, physical interactions between Pol I and both Pol Y1 and Pol Y2 were detected via a yeast two-hybrid assay. It is possible that Pol I plays a role in TLS that is independent of its involvement in DNA repair pathways, but further research is needed to address these questions.

Differing reliance on TLS could also reflect differences in DNA repair pathways in *E. coli* and *B. subtilis*, although many of these pathways are highly conserved [9]. In *E. coli*, DNA lesions produced by 4-NQO, [56] UV, [57] NFZ, [15] and MMC [29] can be repaired by nucleotide excision repair (NER), whereas MMS lesions are repaired by base excision repair (BER) [21]. However, specific repair pathways exist for repair of UV and alkylation damage, and repair can also occur via homologous recombination (HR). In *B. subtilis*, either NER or HR can repair damage caused by 4-NQO and UV, whereas both are required for repair of MMC damage [58]. Like in *E. coli*, NER does not contribute to the repair of MMS damage, but HR does. We are unaware of any direct evidence for which repair pathways respond to NFZ damage in *B. subtilis*, but it would be reasonable to assume that NER is involved, as in *E. coli*. Interestingly, there is evidence that Pol Y1 and Pol Y2 may act in coordination with Pol I in the transcription-coupled NER pathway [10,59]. However, our data reveal no apparent correlations between which pathways repair different lesions and whether Pol Y1, Pol Y2, both, or neither contributes to survival and mutagenesis.

In paired single-molecule imaging experiments, we measured the cellular localization and dynamics of Pol Y1 in untreated cells and upon treatment with the same set of damaging agents. We first characterized the DnaX foci that mark sites of replication. We found that all types of DNA damage led to shifts in the position of DnaX foci toward midcell along the long-cell axis, paired with moderate (25% or less) changes in the average number of foci per cell. The shift in localization of sites of replication sites toward midcell has been observed before and is indicative of perturbations to replication [4,11]. Although the degree of shift varied for different damaging agents, it generally increased in dose-dependent fashion for each damaging agent, as expected. However, despite these obvious perturbations to replication, there were generally only minimal changes in the degree of Pol Y1 colocalization with replication sites, and likewise only minimal changes in Pol Y1 diffusion and binding lifetime.

In *E. coli*, single-molecule imaging studies have suggested a spatial component of Pol IV regulation; in brief, Pol IV is not highly enriched near sites of replication during normal growth, but it is strongly recruited upon DNA damage or other replication perturbations, even for non-cognate lesions or in other situations where Pol IV cannot resolve the challenge to replication [4,6]. Further, DNA damage was found to increase the static fraction of Pol IV molecules by over two-fold, consistent with Pol IV binding near sites of stalled replication and performing TLS; [4] similar behavior has been observed for DNA repair proteins like the polymerase Pol I and ligase, with approximately five-fold increases in the static fraction [50]. Given this model from *E. coli*, it is surprising that we do not observe changes in Pol Y1 dynamics or enrichment at replication sites upon DNA damage, particularly for damaging agents (UV and MMS in this study, and 4-NQO in our previous study [11]) to which it promotes survival. The only exception was NFZ treatment, for which we observed a moderate increase in colocalization for the 50 µM concentration, but not for the higher 100 µM concentration, making interpretation difficult. It is possible that the constitutive association of Pol Y1 with replication forks allows it to act in TLS or in a DNA repair pathway without further enrichment, unlike *E. coli* Pol IV, which is not measurably enriched in the absence of DNA damage.

This study provides new insight into the activity of TLS polymerases in *B. subtilis*, but it also raises new questions. First, what explains the discrepancies between the contributions of Pol Y1 and Pol Y2 to DNA damage survival and mutagenesis? For example, how does Pol Y1 promote survival to MMS without contributing to mutagenesis, and how does Pol Y2 promote MMC-induced mutagenesis while having no impact on survival? Second, are TLS pathways different in vegetative and sporulating cells? A prior study in sporulating cells found effects of Pol Y1 and Pol Y2 deletion on DNA damage survival and mutagenesis after UV and MMC treatment that differ from our results [12]. Related to the molecular mechanisms of TLS, what explains the lack of damage-induced enrichment of Pol Y1 at replication forks and how is it acting to promote cell survival after damage? Both Pol IV [4,60] and Pol Y1 [11] must bind the clamp to perform TLS, and clamp-binding mutations phenocopy a knockout. However, Pol IV is enriched at replication sites upon DNA damage primarily through interactions with the replisome component single-stranded DNA-binding protein (SSB); weakening the Pol IV-SSB interaction leads to a moderate defect in DNA damage tolerance, but a substantial loss in enrichment at the fork [5,6]. It is unknown whether Pol Y1 and Pol Y2 bind SSB; they have not been identified as SSB-interacting proteins via screening, [61] although to our knowledge no study has searched for this interaction specifically. Finally, although Pol Y2 is only present in the cell after SOS induction, [8] does it have similar localization and dynamics to Pol Y1, or is it more strongly recruited to stalled replication forks? We have found that construction of a functional Pol Y2 fusion for microscopy is complicated by its position as the first gene in an operon, but further research should investigate its behavior at the single-molecule level. Given the apparent diversity of bacterial TLS mechanisms, future work is needed to address these questions and to develop a more comprehensive picture of bacterial TLS.

## Materials and methods

### Bacterial strain construction

All bacterial strains used in this study were constructed in the *B. subtilis* WT background PY79 [62,63]. Genetic modifications were introduced by transformation of double-stranded DNA fragments generated by PCR and Gibson assembly or by transformation of genomic DNA, followed by selection on appropriate antibiotic plates. Detailed strain information is provided in the Supplementary Methods, including lists of all oligonucleotides (Table G in S1 Text) and strains (Table H in S1 Text) used in this study.

### DNA damage survival assays

Quantitative survival rates were determined for strains treated with different types and doses of DNA damaging agents following previously reported procedures, [11,13] with slight modifications for different damaging agents. In all cases, strains were first streaked out from glycerol stocks onto LB Lennox agar plates containing an antibiotic for selection. The following day, overnight cultures were inoculated from single colonies and incubated overnight in LB Lennox media shaking at 22 °C. The next morning, fresh cultures were inoculated and grown to exponential phase shaking at 37 °C, then serially diluted and plated on LB Lennox agar plates as described below. After overnight incubation at 37 °C, colonies were enumerated, and the survival rate was calculated. For NFZ treatment, colonies were small and difficult to count accurately after overnight incubation at 37 °C. Thus, plates were left at room temperature for an additional 24 h before counting.

Survival to UV light was assayed exactly as described previously [13] by plating cells on plain LB Lennox agar plates and irradiating with varying doses of 254 nm UV-C light (Analytik Jena UVS-28 EL). Survival to MMS, NFZ, and MMC ware assayed following the same procedure described previously for 4-NQO treatment [11]. In brief, LB Lennox agar plates were prepared containing different concentrations of drug, with the total volume of solvent added held constant across all drug concentrations. MMS was used as a neat liquid, NFZ solutions were prepared in DMF, and MMC solutions were prepared in DMSO.

At least three independent replicates were performed for each damaging agent and checked for consistency. For MMS, we noted higher than normal variability in the survival rates. Therefore, we conducted a total of nine replicates. Inconsistent data points were identified using the MATLAB function isoutlier using the default parameters and were removed from the dataset; in all cases, at least six replicates remained. Although outlier removal reduced the resulting standard deviation in the dataset, it did not have an impact on the qualitative results.

### Rifampicin resistance mutagenesis assays

Mutagenesis was quantified under different treatment conditions by measuring the proportion of cells that were resistant to the antibiotic rifampicin (Rif^R^). These assays were performed following previously reported procedures, [11,13] with slight modifications for different damaging agents. UV mutagenesis assays were performed exactly as described previously, by resuspending cells in transparent MgSO_4_ solution and irradiating with a dose of 40 J/m^2^, then resuspending in fresh LB Lennox media and growing overnight [13]. Mutagenesis assays with MMS, NFZ, and MMC followed the same procedure described previously for 4-NQO treatment [11] but adapted for the different drugs. In brief, each drug was added to liquid culture at final concentrations of 10 mM for MMS (from the neat liquid), 100 µM for NFZ (from a 100 mM stock), and 200 ng/mL for MMC (from a 100 µg/mL stock). Cultures were incubated shaking at 37 °C for 1 h and then washed, resuspended in fresh media, and grown overnight exactly as described previously for 4-NQO mutagenesis assays. For all assays, the overnight cultures were serially diluted and plated on LB Lennox agar plates with or without 10 µg/mL Rif. After overnight incubation, colonies were enumerated, the number of CFUs/mL was determined for Rif^+^ and Rif^−^ plates, and the Rif^R^ frequency was calculated.

At least five independent replicates were performed for each damaging agent and checked for consistency. For each damaging agent, an independent dataset was collected in parallel with untreated cells to measure the frequency of spontaneous rifampicin resistance Rif^R^.

### Cell culture and sample preparation for microscopy

Cell culture and sample preparation for microscopy was performed exactly as described previously [11]. Imaging cultures were grown in minimal S7_50_-sorbitol media at 25 mL scale shaking at 37 °C. Samples were harvested in early exponential phase (OD_600nm_ ≈ 0.15 for untreated samples), labeled with JFX_554_ dye at a final concentration of 2.5 nM, and incubated shaking at 37 °C for 15 min. After labeling, cells were washed, concentrated by centrifugation, deposited on an agarose pad containing growth media, and sandwiched between two cleaned coverslips. For P_*yneA*_*-gfp* SOS reporter imaging, cells were harvested and concentrated by a single centrifugation step, then imaged directly without labeling.

### Sample treatment for microscopy

Cultures were treated with DNA damaging agents when they reached the same OD_600nm_ ≈ 0.15. For UV treatment, the entire culture was transferred to a sterile Petri dish and irradiated with 254 nm UV-C light (Analytik Jena UVS-28 EL) with gentle stirring. An aliquot was taken for labeling and imaging (the *t* = 0 h sample) and the remaining culture was returned to a culture tube and grown for an additional hour shaking at 37 °C, at which point a second aliquot was taken for labeling and imaging (the *t* = 1 h sample). Treatment with MMS, NFZ, and MMC was performed following the same procedure described previously for 4-NQO treatment [11]. In brief, the drug was added as a neat liquid (for MMS) or from a stock solution at a 1:1,000 dilution (for NFZ and MMC) to the culture, after which the culture was grown for an additional hour before a sample was harvested for labeling and imaging.

For all treatment conditions, the effect on cell survival was quantified as described previously for 4-NQO treatment [11]. Aliquots were taken before and after treatment, serially diluted, and plated on plain LB Lennox agar plates. The number of CFUs/mL was determined for treated and untreated samples after overnight incubation and the fold-changed was calculated.

### Microscopy

Microscopy was performed using a custom fluorescence microscope described previously [11]. A Nikon Ti2-E inverted microscope was equipped with a Nikon CFI Apo 100 × /1.49 NA total internal reflection fluorescence (TIRF) objective lens and Chroma filters. Laser excitation (Coherent Sapphire) at 514 nm (∼1 W cm^−2^ power density) and 561 nm (∼15 W cm^−2^ power density) was used to excite DnaX-mYPet and Pol Y1-Halo-JFX_554_ fluorescence, respectively. P_*yneA*_*-gfp* SOS reporter imaging used 514 nm excitation (~ 15 W cm^−2^ power density). Movies were recorded with a Hamamatsu ImageEM C9100-23BKIT EMCCD camera. Highly inclined thin illumination, or near-TIRF, illumination was achieved by focusing the laser light to the back focal plane of the objective [64]. Brightfield images of cells were recorded for each field of view using white light transillumination. For localization, colocalization, and diffusion analyses, movies were recorded using a short integration time (13.9 ms) to enable detection of both mobile and static Pol Y1 molecules. This short integration time was also used for P_*yneA*_*-gfp* SOS reporter imaging. For binding lifetime analysis, a longer integration time (250 ms) was used for selective detection of static molecules [11].

### Image and data analysis

Quantitative image analysis was performed exactly as described previously [11]. In brief, the MATLAB package MicrobeTracker [65] was used for cell segmentation of brightfield images, and the MATLAB package u-track [66,67] was used for spot detection and tracking of fluorescence movies. For analysis of DnaX-mYPet foci, the first 20 frames of 514 nm excitation were averaged.

Likewise, the same analysis approaches and custom code were used for determination of average DnaX and Pol Y1 cellular localization, Pol Y1-DnaX colocalization (through radial distribution function analysis), Pol Y1 diffusion (using the MSD approach to determine the apparent diffusion coefficient D^*^), and Pol Y1 binding lifetime [11]. Localization and colocalization analyses were performed for all trajectories with at least five localizations and diffusion analysis was performed for all trajectories with at least four steps. Binding lifetime analysis was also performed as described previously [11]. In brief, apparent binding lifetimes were determined by single-exponential fits to the distribution of trajectory lengths, excluding tracks less than five frames in length. Apparent binding lifetimes were corrected for photobleaching using the photobleaching lifetime measured previously (τ_Bleach_ = 1.10 ± 0.05 s), and both apparent and corrected lifetimes are reported. Cell morphology was determined from MicrobeTracker cell segmentation output.

The D^*^ distributions were fit to a 3-state model, with a static DNA-bound population, a highly mobile population, and a population with intermediate mobility, which may reflect molecules binding transiently to DNA. We note that visual comparison of an alternative 2-state model fit indicated that it could not describe the data well, as reported in our prior study [11]. To validate this choice statistically, we compared the Akaike Information Criterion (AIC) values for 2-state vs. 3-state fits; this metric reflects goodness of fit while penalizing model complexity. Smaller AIC values indicate a better fit; when the AIC values are negative, more negative values indicate a better fit. For all diffusion datasets, the AIC values for the 3-state model were smaller than those for the 2-state model, confirming that the 3-state model better describes the data. As an example, for the untreated cell diffusion data shown in Fig 6A, the AIC value is -52.7 for the 2-state fit and -127.8 for the 3-state fit.

For analysis of P_*yneA*_*-gfp* SOS reporter intensity, the first frame of the fluorescence movie was analyzed. Pixel intensity was corrected by subtraction of the background camera offset value, and then the average background-subtracted intensity was calculated for each cell from all pixels within the cell outline.

All imaging experiments were performed on at least two separate days with at least three separate replicates, defined as separate imaging cultures, with the exception of P_*yneA*_*-gfp* SOS reporter imaging, for which only two replicates were obtained. The exact number of imaging days, replicates, cells, and tracks or foci for all Figures are listed in Table I in S1 Text.

For survival, mutagenesis, and imaging datasets, statistical comparisons were made using the two-tailed Wilcoxon rank sum test, implemented in the MATLAB function ranksum. For significance testing of survival datasets, the survival rate for each mutant strain was compared to that of the WT strain at each individual treatment condition (*i.e.*, at each UV dose or drug concentration). The Benjamini-Hochberg procedure, implemented in the MATLAB function mafdr, was used to correct for multiple comparisons between mutant strains and the WT strain (for survival and mutagenesis assays) or between untreated and treated cells (for imaging data). Corrected *p*-values were assessed for significance at the *p* < 0.05 level.

## Supporting information

S1 Fig Characterization of SOS induction upon DNA damage. Average fluorescence intensity of cells bearing the P_*yneA*_*-gfp* SOS reporter for untreated cells or cells treated with 40 J/m^2^ 254 nm UV light, 10 mM MMS, 100 µM NFZ, and 200 ng/mL MMC. The red lines indicate the median, the boxes represent the interquartile range, and the whiskers encompass the rest of the data range. Individual outliers are shown as red + signs. (Note that the *y*-axis was truncated to show the comparison between the conditions more clearly, but a few outliers for UV treatment fall outside the plotted region.)
(TIF)

S2 Fig Characterization of Pol Y1-HaloTag fusion functionality. Relative survival rates for WT, ΔPol Y1 knockout, Pol Y1-Halo fusion, and Pol Y1-Halo plus DnaX-mYPet fusion strains after treatment with different doses of 254 nm UV light. Note that the *y*-axis is on a log scale.
(TIF)

S3 Fig Average cellular localization of DnaX-mYPet and Pol Y1-Halo in the presence of different types of DNA damage. Short axis projections of DnaX foci (left) and Pol Y1 trajectories (right) in **(A)** untreated cells and after treatment with **(B)** 10 J/m^2^ 254 nm UV light (*t* = 0 h), **(C)** 10 J/m^2^ 254 nm UV light (*t* = 1 h), **(D)** 20 J/m^2^ 254 nm UV light (*t* = 0 h), **(E)** 20 J/m^2^ 254 nm UV light (*t* = 1 h), **(F)** 5 mM MMS, **(G)** 10 mM MMS, **(H)** 15 mM MMS, **(I)** 100 µM NFZ, **(J)** 100 µM NFZ, **(K)** 50 ng/mL MMC, and **(L)** 100 ng/mL MMC.
(TIF)

S4 Fig Average cellular localization of DnaX-mYPet and Pol Y1-Halo in the presence of different types of DNA damage. Long axis projections of DnaX foci (left) and Pol Y1 trajectories (right) after treatment with **(A)** 20 J/m^2^ 254 nm UV light (*t* = 0 h), **(B)** 20 J/m^2^ 254 nm UV light (*t* = 1 h), **(C)** 10 mM MMS, **(D)** 15 mM MMS, **(E)** 50 µM NFZ, and **(F)** 100 ng/mL MMC. The corresponding short axis projections are shown in S3 Fig.
(TIF)

S5 Fig Apparent diffusion coefficient (D^*^) distributions and corresponding three-population fits for Pol Y1-Halo in the presence of different types of DNA damage. Pol Y1 D^*^ distributions in after treatment with **(A)** 20 J/m^2^ 254 nm UV light (*t* = 0 h), **(B)** 20 J/m^2^ 254 nm UV light (*t* = 1 h), **(C)** 10 mM MMS, **(D)** 15 mM MMS, **(E)** 100 µM NFZ, and **(F)** 100 ng/mL MMC. Individual populations are shown as red dashed lines and overall fits are shown as solid black lines.
(TIF)

S6 Fig Pol Y1-Halo binding lifetime measurements in the presence of different types of DNA damage. Distributions of apparent binding lifetime and corresponding exponential fits for (A) untreated cells and after treatment with (B) 10 J/m^2^ 254 nm UV light (*t* = 0 h), (C) 10 J/m^2^ 254 nm UV light (*t* = 1 h), (D) 20 J/m^2^ 254 nm UV light (*t* = 0 h), (E) 20 J/m^2^ 254 nm UV light (*t* = 1 h), (F) 5 mM MMS, (G) 10 mM MMS, (H) 15 mM MMS, (I) 100 µM NFZ, (J) 100 µM NFZ, (K) 50 ng/mL MMC, and (L) 100 ng/mL MMC. (Note that the *y*-axes are truncated to show the longer timescale behavior more clearly.).
(TIF)

S1 Text Supporting Information. Supplementary Tables A – I and Supplementary Methods.
(PDF)

## References

[1] Fuchs RP, Fujii S. Translesion DNA synthesis and mutagenesis in prokaryotes. Cold spring harbor perspectives in biology. 2013 Dec 1;5(12):a012682–a012682. doi: 10.1101/cshperspect.a012682 24296168 PMC3839610

[2] Fujii S, Fuchs RP. A comprehensive view of translesion synthesis in *Escherichia coli*. Microbiol Mol Biol Rev. 2020;84(3):e00002-20. doi: 10.1128/MMBR.00002-20 32554755 PMC7307797

[3] Joseph AM, Badrinarayanan A. Visualizing mutagenic repair: Novel insights into bacterial translesion synthesis. FEMS Microbiol Rev. 2020;44(5):572–82. doi: 10.1093/femsre/fuaa023 32556198 PMC7476773

[4] Thrall ES, Kath JE, Chang S, Loparo JJ. Single-molecule imaging reveals multiple pathways for the recruitment of translesion polymerases after DNA damage. Nat Commun. 2017;8(1):2170. doi: 10.1038/s41467-017-02333-2 29255195 PMC5735139

[5] Chang S, Thrall ES, Laureti L, Piatt SC, Pagès V, Loparo JJ. Compartmentalization of the replication fork by single-stranded DNA-binding protein regulates translesion synthesis. Nat Struct Mol Biol. 2022;29(9):932–41. doi: 10.1038/s41594-022-00827-2 36127468 PMC9509481

[6] Thrall ES, Piatt SC, Chang S, Loparo JJ. Replication stalling activates SSB for recruitment of DNA damage tolerance factors. Proc Natl Acad Sci USA. 2022;119(41):e2208875119. doi: 10.1073/pnas.2208875119 36191223 PMC9565051

[7] Sung H-M, Yeamans G, Ross CA, Yasbin RE. Roles of YqjH and YqjW, homologs of the Escherichia coli UmuC/DinB or Y superfamily of DNA polymerases, in stationary-phase mutagenesis and UV-induced mutagenesis of Bacillus subtilis. J Bacteriol. 2003;185(7):2153–60. doi: 10.1128/JB.185.7.2153-2160.2003 12644484 PMC151490

[8] Duigou S, Ehrlich SD, Noirot P, Noirot-Gros M-F. Distinctive genetic features exhibited by the Y-family DNA polymerases in *Bacillus subtilis*. Mol Microbiol. 2004;54(2):439–51. doi: 10.1111/j.1365-2958.2004.04259.x 15469515

[9] Lenhart JS, Schroeder JW, Walsh BW, Simmons LA. DNA repair and genome maintenance in *Bacillus subtilis*. Microbiol Mol Biol Rev. 2012;76(3):530–64. doi: 10.1128/MMBR.05020-11 22933559 PMC3429619

[10] Million-Weaver S, Samadpour AN, Moreno-Habel DA, Nugent P, Brittnacher MJ, Weiss E, et al. An underlying mechanism for the increased mutagenesis of lagging-strand genes in *Bacillus subtilis*. Proc Natl Acad Sci U S A. 2015;112(10):E1096-105. doi: 10.1073/pnas.1416651112 25713353 PMC4364195

[11] Marrin ME, Foster MR, Santana CM, Choi Y, Jassal AS, Rancic SJ, et al. The translesion polymerase Pol Y1 is a constitutive component of the B. subtilis replication machinery. Nucleic Acids Res. 2024;52(16):9613–29. doi: 10.1093/nar/gkae637 39051562 PMC11381352

[12] Rivas-Castillo AM, Yasbin RE, Robleto E, Nicholson WL, Pedraza-Reyes M. Role of the Y-family DNA polymerases YqjH and YqjW in protecting sporulating *Bacillus subtilis* cells from DNA damage. Curr Microbiol. 2010;60(4):263–7. doi: 10.1007/s00284-009-9535-3 19924481

[13] O’Neal LG, Drucker MN, Lai NK, Clemente AF, Campbell AP, Way LE, et al. The B. subtilis replicative polymerases bind the sliding clamp with different strengths to tune their activity in DNA replication. Nucleic Acids Research. 2025;53(14):gkaf721. doi: 10.1093/nar/gkaf721 40737090 PMC12309361

[14] Courcelle CT, Belle JJ, Courcelle J. Nucleotide excision repair or polymerase V-mediated lesion bypass can act to restore UV-arrested replication forks in *Escherichia coli*. J Bacteriol. 2005;187(20):6953–61. doi: 10.1128/JB.187.20.6953-6961.2005 16199565 PMC1251618

[15] Ona KR, Courcelle CT, Courcelle J. Nucleotide excision repair is a predominant mechanism for processing nitrofurazone-induced DNA damage in *Escherichia coli*. J Bacteriol. 2009;191(15):4959–65. doi: 10.1128/JB.00495-09 19465649 PMC2715711

[16] Santos-Escobar F, Leyva-Sánchez HC, Ramírez-Ramírez N, Obregón-Herrera A, Pedraza-Reyes M. Roles of Bacillus subtilis RecA, nucleotide excision repair, and translesion synthesis polymerases in counteracting Cr(VI)-promoted DNA damage. J Bacteriol. 2019;201(8):e00073-19. doi: 10.1128/JB.00073-19 30745368 PMC6436346

[17] Au N, Kuester-Schoeck E, Mandava V, Bothwell LE, Canny SP, Chachu K, et al. Genetic composition of the Bacillus subtilis SOS system. J Bacteriol. 2005;187(22):7655–66. doi: 10.1128/JB.187.22.7655-7666.2005 16267290 PMC1280312

[18] Sinha RP, Häder DP. UV-induced DNA damage and repair: A review. Photochem Photobiol Sci. 2002;1(4):225–36. doi: 10.1039/b201230h 12661961

[19] Tang M, Pham P, Shen X, Taylor JS, O’Donnell M, Woodgate R, et al. Roles of *E. coli* DNA polymerases IV and V in lesion-targeted and untargeted SOS mutagenesis. Nature. 2000;404(6781):1014–8. doi: 10.1038/35010020 10801133

[20] Wyatt MD, Pittman DL. Methylating agents and DNA repair responses: Methylated bases and sources of strand breaks. Chem Res Toxicol. 2006;19(12):1580–94. doi: 10.1021/tx060164e 17173371 PMC2542901

[21] Sikora A, Mielecki D, Chojnacka A, Nieminuszczy J, Wrzesinski M, Grzesiuk E. Lethal and mutagenic properties of MMS-generated DNA lesions in *Escherichia coli* cells deficient in BER and AlkB-directed DNA repair. Mutagenesis. 2010;25(2):139–47. doi: 10.1093/mutage/gep052 19892776

[22] Scotland MK, Heltzel JMH, Kath JE, Choi JS, Berdis AJ, Loparo JJ. A genetic selection for dinB mutants reveals an interaction between DNA polymerase IV and the replicative polymerase that is required for translesion synthesis. PLoS Genet. 2015;11(9):e1005507. doi: 10.1371/journal.pgen.1005507 26352807 PMC4564189

[23] McCalla DR, Reuvers A, Kaiser C. Mode of action of nitrofurazone. J Bacteriol. 1970;104(3):1126–34. doi: 10.1128/jb.104.3.1126-1134.1970 16559085 PMC248269

[24] Tu Y, McCalla DR. Effect of activated nitrofurans on DNA. Biochim Biophys Acta. 1975;402(2):142–9. doi: 10.1016/0005-2787(75)90032-5 1100114

[25] Bjedov I, Dasgupta CN, Slade D, Le Blastier S, Selva M, Matic I. Involvement of *Escherichia coli* DNA polymerase IV in tolerance of cytotoxic alkylating DNA lesions in vivo. Genetics. 2007;176(3):1431–40. doi: 10.1534/genetics.107.072405 17483416 PMC1931539

[26] Iyer VN, Szybalski W. A molecular mechanism of mitomycin action: Linking of complementary dna strands. Proc Natl Acad Sci U S A. 1963;50(2):355–62. doi: 10.1073/pnas.50.2.355 14060656 PMC221180

[27] Tomasz M. The Mitomycins: Natural Cross-linkers of DNA. Molecular Aspects of Anticancer Drug-DNA Interactions. Macmillan Education UK. 1994. 312–49. doi: 10.1007/978-1-349-13330-7_8

[28] Tomasz M, Palom Y. The mitomycin bioreductive antitumor agents: Cross-linking and alkylation of DNA as the molecular basis of their activity. Pharmacol Ther. 1997;76(1–3):73–87. doi: 10.1016/s0163-7258(97)00088-0 9535170

[29] Dronkert ML, Kanaar R. Repair of DNA interstrand cross-links. Mutat Res. 2001;486(4):217–47. doi: 10.1016/s0921-8777(01)00092-1 11516927

[30] Gawel D, Maliszewska-Tkaczyk M, Jonczyk P, Schaaper RM, Fijalkowska IJ. Lack of strand bias in UV-induced mutagenesis in Escherichia coli. J Bacteriol. 2002;184(16):4449–54. doi: 10.1128/JB.184.16.4449-4454.2002 12142415 PMC135265

[31] Benson RW, Norton MD, Lin I, Du Comb WS, Godoy VG. An active site aromatic triad in *Escherichia coli* DNA Pol IV coordinates cell survival and mutagenesis in different DNA damaging agents. PLoS One. 2011;6(5):e19944. doi: 10.1371/journal.pone.0019944 21614131 PMC3096655

[32] Jarosz DF, Godoy VG, Delaney JC, Essigmann JM, Walker GC. A single amino acid governs enhanced activity of DinB DNA polymerases on damaged templates. Nature. 2006;439(7073):225–8. doi: 10.1038/nature04318 16407906

[33] Dupes NM, Walsh BW, Klocko AD, Lenhart JS, Peterson HL, Gessert DA, et al. Mutations in the *Bacillus subtilis* beta clamp that separate its roles in DNA replication from mismatch repair. J Bacteriol. 2010;192(13):3452–63. doi: 10.1128/JB.01435-09 20453097 PMC2897676

[34] Li Y, Chen Z, Matthews LA, Simmons LA, Biteen JS. Dynamic exchange of two essential DNA Polymerases during replication and after fork arrest. Biophys J. 2019;116(4):684–93. doi: 10.1016/j.bpj.2019.01.008 30686488 PMC6382952

[35] Gozzi K, Ching C, Paruthiyil S, Zhao Y, Godoy-Carter V, Chai Y. Bacillus subtilis utilizes the DNA damage response to manage multicellular development. NPJ Biofilms Microbiomes. 2017;3:8. doi: 10.1038/s41522-017-0016-3 28649409 PMC5445613

[36] Love PE, Lyle MJ, Yasbin RE. DNA-damage-inducible (din) loci are transcriptionally activated in competent *Bacillus subtilis*. Proc Natl Acad Sci U S A. 1985;82(18):6201–5. doi: 10.1073/pnas.82.18.6201 3929251 PMC391020

[37] Nguyen AW, Daugherty PS. Evolutionary optimization of fluorescent proteins for intracellular FRET. Nat Biotechnol. 2005;23(3):355–60. doi: 10.1038/nbt1066 15696158

[38] Wang X, Montero Llopis P, Rudner DZ. Bacillus subtilis chromosome organization oscillates between two distinct patterns. Proc Natl Acad Sci U S A. 2014;111(35):12877–82. doi: 10.1073/pnas.1407461111 25071173 PMC4156703

[39] Liao Y, Schroeder JW, Gao B, Simmons LA, Biteen JS. Single-molecule motions and interactions in live cells reveal target search dynamics in mismatch repair. Proc Natl Acad Sci U S A. 2015;112(50):E6898-906. doi: 10.1073/pnas.1507386112 26575623 PMC4687589

[40] Mangiameli SM, Veit BT, Merrikh H, Wiggins PA. The replisomes remain spatially proximal throughout the cell cycle in bacteria. PLoS Genet. 2017;13(1):e1006582. doi: 10.1371/journal.pgen.1006582 28114307 PMC5293282

[41] Hernández-Tamayo R, Oviedo-Bocanegra LM, Fritz G, Graumann PL. Symmetric activity of DNA polymerases at and recruitment of exonuclease ExoR and of PolA to the Bacillus subtilis replication forks. Nucleic Acids Res. 2019;47(16):8521–36. doi: 10.1093/nar/gkz554 31251806 PMC6895272

[42] Grimm JB, Xie L, Casler JC, Patel R, Tkachuk AN, Falco N, et al. A general method to improve fluorophores using deuterated auxochromes. JACS Au. 2021;1(5):690–6. doi: 10.1021/jacsau.1c00006 34056637 PMC8154212

[43] Soubry N, Wang A, Reyes-Lamothe R. Replisome activity slowdown after exposure to ultraviolet light in *Escherichia coli*. Proc Natl Acad Sci U S A. 2019;116(24):11747–53. doi: 10.1073/pnas.1819297116 31127046 PMC6575178

[44] Uphoff S. Real-time dynamics of mutagenesis reveal the chronology of DNA repair and damage tolerance responses in single cells. Proc Natl Acad Sci USA. 2018;115(28). doi: 10.1073/pnas.1801101115 29941584 PMC6048535

[45] Hernández-Tamayo R, Graumann PL. Bacillus subtilis RarA forms damage-inducible foci that scan the entire cell. BMC Res Notes. 2019;12(1):219. doi: 10.1186/s13104-019-4252-x 30971308 PMC6458690

[46] Mallik S, Popodi EM, Hanson AJ, Foster PL. Interactions and localization of *Escherichia coli* error-prone DNA polymerase IV after DNA damage. J Bacteriol. 2015;197(17):2792–809. doi: 10.1128/JB.00101-15 26100038 PMC4524043

[47] Klocko AD, Schroeder JW, Walsh BW, Lenhart JS, Evans ML, Simmons LA. Mismatch repair causes the dynamic release of an essential DNA polymerase from the replication fork. Mol Microbiol. 2011;82(3):648–63. doi: 10.1111/j.1365-2958.2011.07841.x 21958350 PMC4260453

[48] Hernández-Tamayo R, Schmitz H, Graumann PL. Single-molecule dynamics at a bacterial replication fork after nutritional downshift or chemically induced block in replication. Bowman GR. mSphere. 2021;6(1):e00948-20. doi: 10.1128/mSphere.00948-20 33504660 PMC7885319

[49] Zawadzki P, Stracy M, Ginda K, Zawadzka K, Lesterlin C, Kapanidis AN, et al. The localization and action of topoisomerase IV in *Escherichia coli* chromosome segregation is coordinated by the SMC complex, MukBEF. Cell Rep. 2015;13(11):2587–96. doi: 10.1016/j.celrep.2015.11.034 26686641 PMC5061553

[50] Uphoff S, Reyes-Lamothe R, Garza de Leon F, Sherratt DJ, Kapanidis AN. Single-molecule DNA repair in live bacteria. Proc Natl Acad Sci U S A. 2013;110(20):8063–8. doi: 10.1073/pnas.1301804110 23630273 PMC3657774

[51] Lepore A, Thédié D, McLaren L, Goossens L, Azeroglu B, Pambos OJ, et al. In vivo single-molecule imaging of RecB reveals efficient repair of DNA damage in Escherichia coli. Nucleic Acids Res. 2025;53(10):gkaf454. doi: 10.1093/nar/gkaf454 40464683 PMC12135197

[52] Williams AB, Hetrick KM, Foster PL. Interplay of DNA repair, homologous recombination, and DNA polymerases in resistance to the DNA damaging agent 4-nitroquinoline-1-oxide in Escherichia coli. DNA Repair (Amst). 2010;9(10):1090–7. doi: 10.1016/j.dnarep.2010.07.008 20724226 PMC2949549

[53] Le Chatelier E, Bécherel OJ, d’Alençon E, Canceill D, Ehrlich SD, Fuchs RPP, et al. Involvement of DnaE, the second replicative DNA polymerase from *Bacillus subtilis*, in DNA mutagenesis. J Biol Chem. 2004;279(3):1757–67. doi: 10.1074/jbc.M310719200 14593098

[54] Bruck I, Goodman MF, O’Donnell M. The essential C family DnaE polymerase is error-prone and efficient at lesion bypass. Journal of Biological Chemistry. 2003;278(45):44361–8. doi: 10.1074/jbc.M308307200 12949067

[55] Duigou S, Ehrlich SD, Noirot P, Noirot-Gros M-F. DNA polymerase I acts in translesion synthesis mediated by the Y-polymerases in *Bacillus subtilis*. Mol Microbiol. 2005;57(3):678–90. doi: 10.1111/j.1365-2958.2005.04725.x 16045613

[56] Ikenaga M, Ichikawa-Ryo H, Kondo S. The major cause of inactivation and mutation by 4-nitroquinoline 1-oixde in *Escherichia coli*: excisable 4NQO-purine adducts. J Mol Biol. 1975;92(2):341–56. doi: 10.1016/0022-2836(75)90233-8 806692

[57] Goosen N, Moolenaar GF. Repair of UV damage in bacteria. DNA Repair (Amst). 2008;7(3):353–79. doi: 10.1016/j.dnarep.2007.09.002 17951115

[58] Friedman BM, Yasbin RE. The genetics and specificity of the constitutive excision repair system of *Bacillus subtilis*. Mol Gen Genet. 1983;190(3):481–6. doi: 10.1007/BF00331080 6410154

[59] Carvajal-Garcia J, Samadpour AN, Hernandez Viera AJ, Merrikh H. Oxidative stress drives mutagenesis through transcription-coupled repair in bacteria. Proc Natl Acad Sci USA. 2023;120(27):e2300761120. doi: 10.1073/pnas.2300761120 37364106 PMC10318952

[60] Becherel OJ, Fuchs RPP, Wagner J. Pivotal role of the beta-clamp in translesion DNA synthesis and mutagenesis in *E. coli* cells. DNA Repair (Amst). 2002;1(9):703–8. doi: 10.1016/s1568-7864(02)00106-4 12509274

[61] Costes A, Lecointe F, McGovern S, Quevillon-Cheruel S, Polard P. The C-terminal domain of the bacterial SSB protein acts as a DNA maintenance Hub at active chromosome replication forks. PLoS Genet. 2010;6(12):e1001238. doi: 10.1371/journal.pgen.1001238 21170359 PMC3000357

[62] Zeigler DR, Prágai Z, Rodriguez S, Chevreux B, Muffler A, Albert T, et al. The origins of 168, W23, and other *Bacillus subtilis* legacy strains. J Bacteriol. 2008;190(21):6983–95. doi: 10.1128/JB.00722-08 18723616 PMC2580678

[63] Schroeder JW, Simmons LA. Complete genome sequence of *Bacillus subtilis* Strain PY79. Genome Announc. 2013;1(6):e01085-13. doi: 10.1128/genomeA.01085-13 24356846 PMC3868870

[64] Tokunaga M, Imamoto N, Sakata-Sogawa K. Highly inclined thin illumination enables clear single-molecule imaging in cells. Nat Methods. 2008;5(2):159–61. doi: 10.1038/nmeth1171 18176568

[65] Sliusarenko O, Heinritz J, Emonet T, Jacobs-Wagner C. High-throughput, subpixel precision analysis of bacterial morphogenesis and intracellular spatio-temporal dynamics. Mol Microbiol. 2011;80(3):612–27. doi: 10.1111/j.1365-2958.2011.07579.x 21414037 PMC3090749

[66] Jaqaman K, Loerke D, Mettlen M, Kuwata H, Grinstein S, Schmid SL, et al. Robust single-particle tracking in live-cell time-lapse sequences. Nat Methods. 2008;5(8):695–702. doi: 10.1038/nmeth.1237 18641657 PMC2747604

[67] Aguet F, Antonescu CN, Mettlen M, Schmid SL, Danuser G. Advances in analysis of low signal-to-noise images link dynamin and AP2 to the functions of an endocytic checkpoint. Dev Cell. 2013;26(3):279–91. doi: 10.1016/j.devcel.2013.06.019 23891661 PMC3939604

