# Supplementary figures and images for: The B. subtilis translesion polymerase Pol Y1 is not strongly recruited to sites of replication upon different types of DNA damage
Source: PLoS Genet. 2026 Jul 14;22(7):e1012246. doi: 10.1371/journal.pgen.1012246 (PMC13387608; doi:10.1371/journal.pgen.1012246)

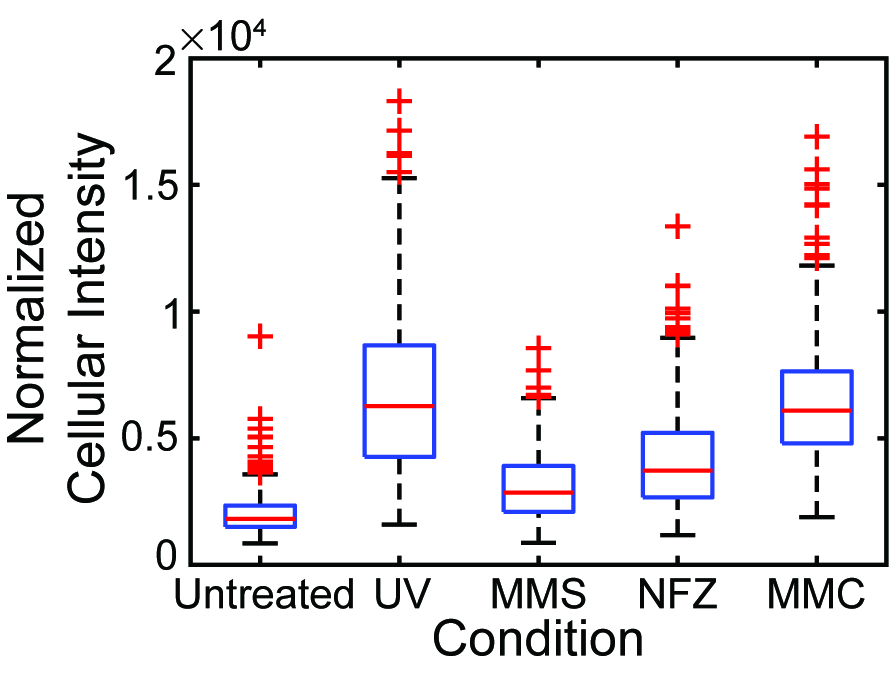

Supplement: S1 Fig — Average fluorescence intensity of cells bearing the PyneA-gfp SOS reporter for untreated cells or cells treated with 40 J/m2 254 nm UV light, 10 mM MMS, 100 µM NFZ, and 200 ng/mL MMC. The red lines indicate the median, the boxes represent the interquartile range, and the whiskers encompass the rest of the data range. Individual outliers are shown as red + signs. (Note that the y-axis was truncated to show the comparison between the conditions more clearly, but a few outliers for UV treatment fall outside the plotted region.) (TIF) [file pgen.1012246.s001.tif]

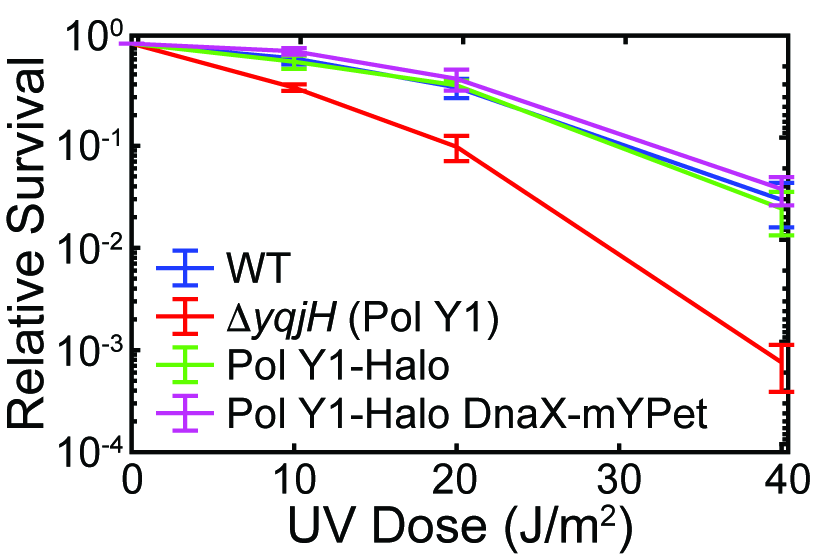

Supplement: S2 Fig — Relative survival rates for WT, ΔPol Y1 knockout, Pol Y1-Halo fusion, and Pol Y1-Halo plus DnaX-mYPet fusion strains after treatment with different doses of 254 nm UV light. Note that the y-axis is on a log scale. (TIF) [file pgen.1012246.s002.tif]

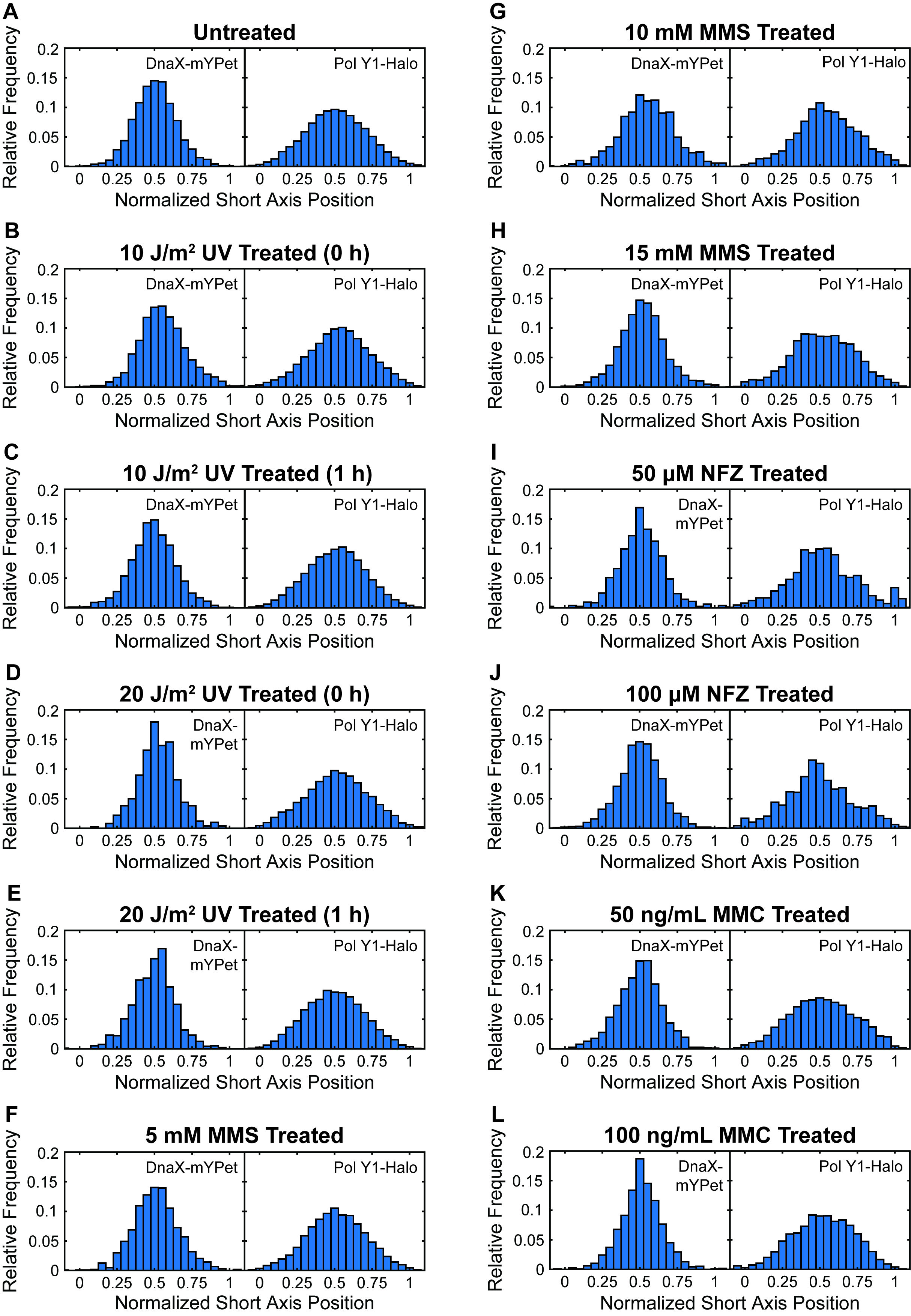

Supplement: S3 Fig — Short axis projections of DnaX foci (left) and Pol Y1 trajectories (right) in (A) untreated cells and after treatment with (B) 10 J/m2 254 nm UV light (t = 0 h), (C) 10 J/m2 254 nm UV light (t = 1 h), (D) 20 J/m2 254 nm UV light (t = 0 h), (E) 20 J/m2 254 nm UV light (t = 1 h), (F) 5 mM MMS, (G) 10 mM MMS, (H) 15 mM MMS, (I) 100 µM NFZ, (J) 100 µM NFZ, (K) 50 ng/mL MMC, and (L) 100 ng/mL MMC. (TIF) [file pgen.1012246.s003.tif]

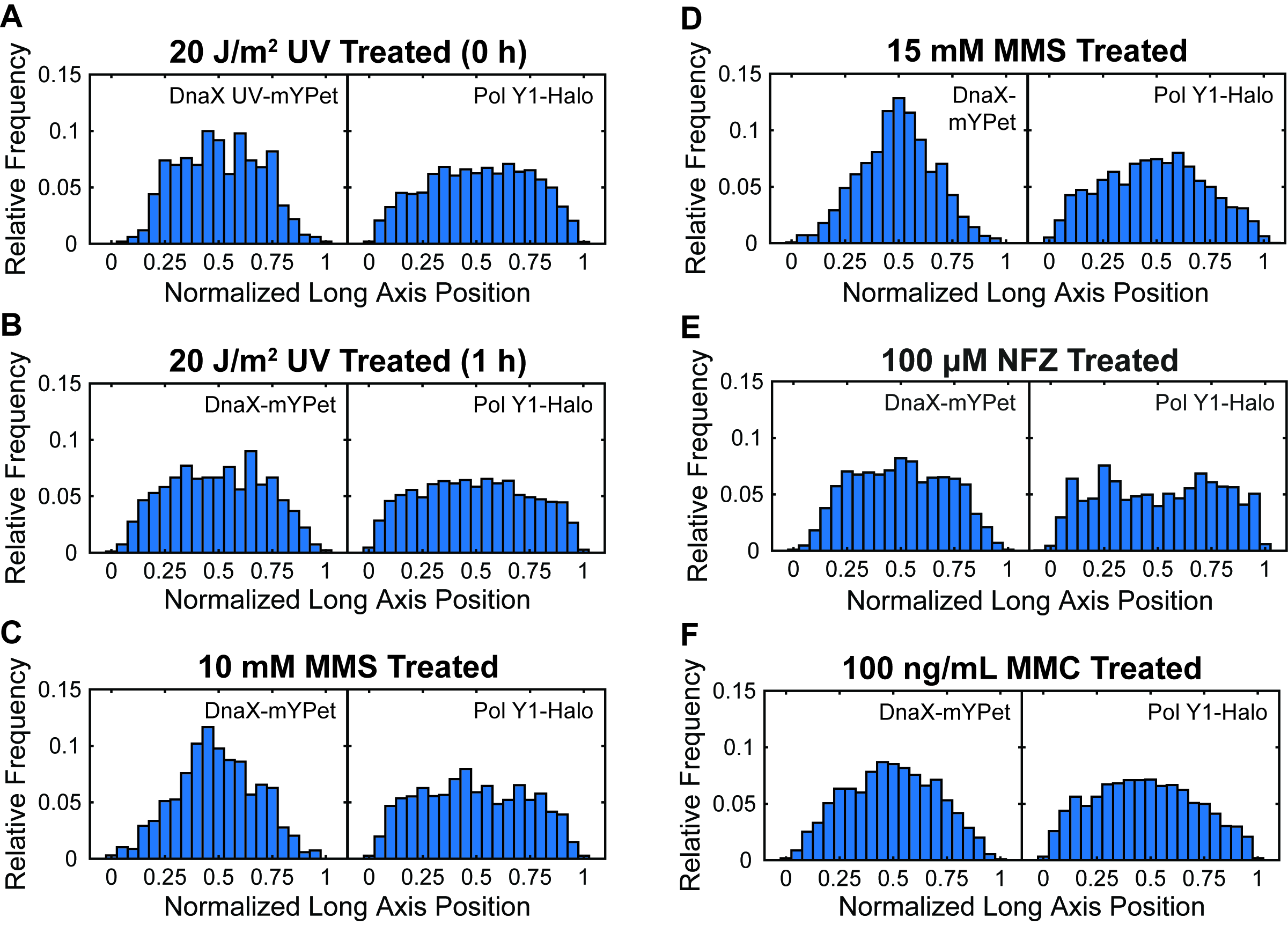

Supplement: S4 Fig — Long axis projections of DnaX foci (left) and Pol Y1 trajectories (right) after treatment with (A) 20 J/m2 254 nm UV light (t = 0 h), (B) 20 J/m2 254 nm UV light (t = 1 h), (C) 10 mM MMS, (D) 15 mM MMS, (E) 50 µM NFZ, and (F) 100 ng/mL MMC. The corresponding short axis projections are shown in S3 Fig. (TIF) [file pgen.1012246.s004.tif]

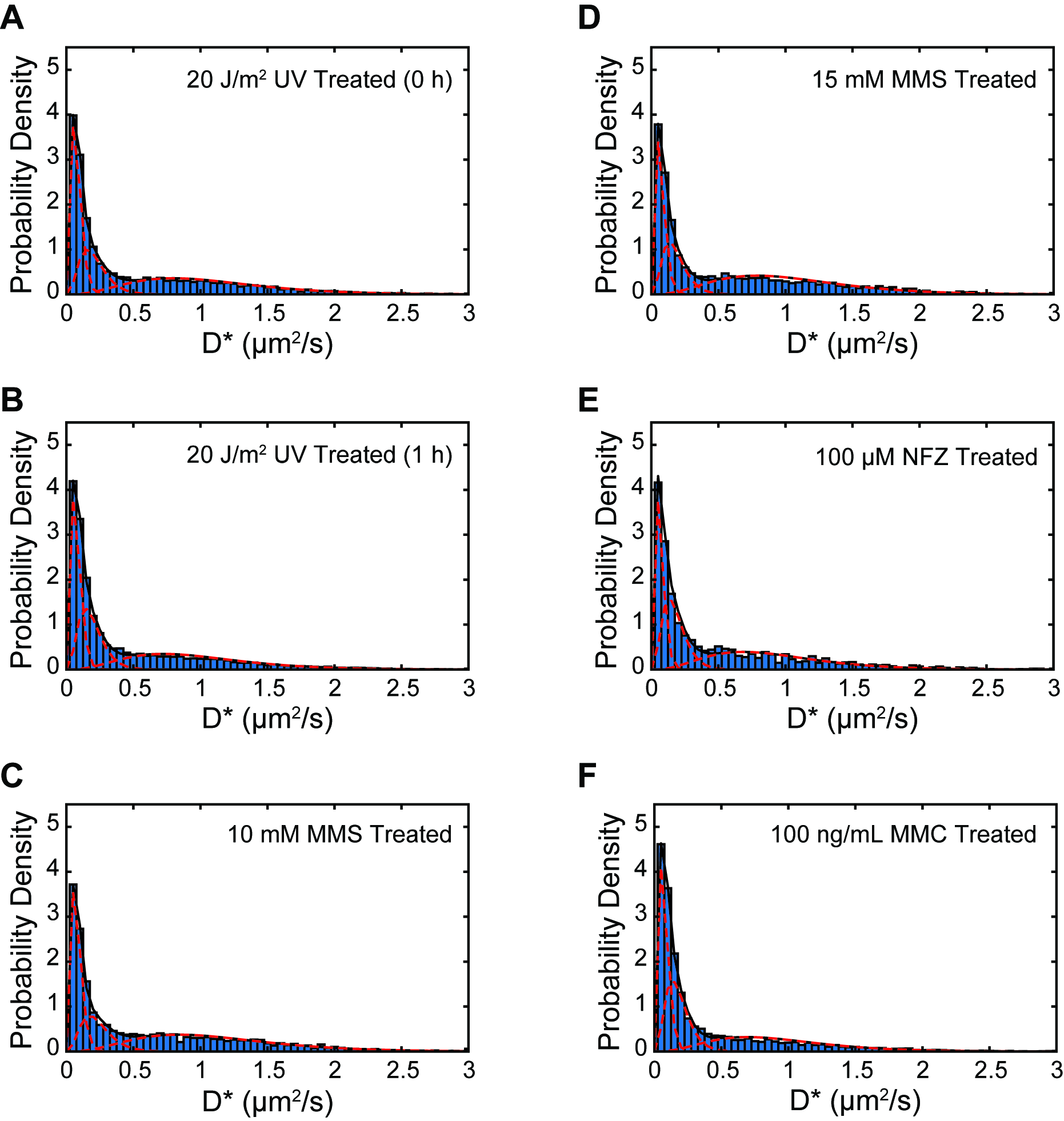

Supplement: S5 Fig — Pol Y1 D* distributions in after treatment with (A) 20 J/m2 254 nm UV light (t = 0 h), (B) 20 J/m2 254 nm UV light (t = 1 h), (C) 10 mM MMS, (D) 15 mM MMS, (E) 100 µM NFZ, and (F) 100 ng/mL MMC. Individual populations are shown as red dashed lines and overall fits are shown as solid black lines. (TIF) [file pgen.1012246.s005.tif]

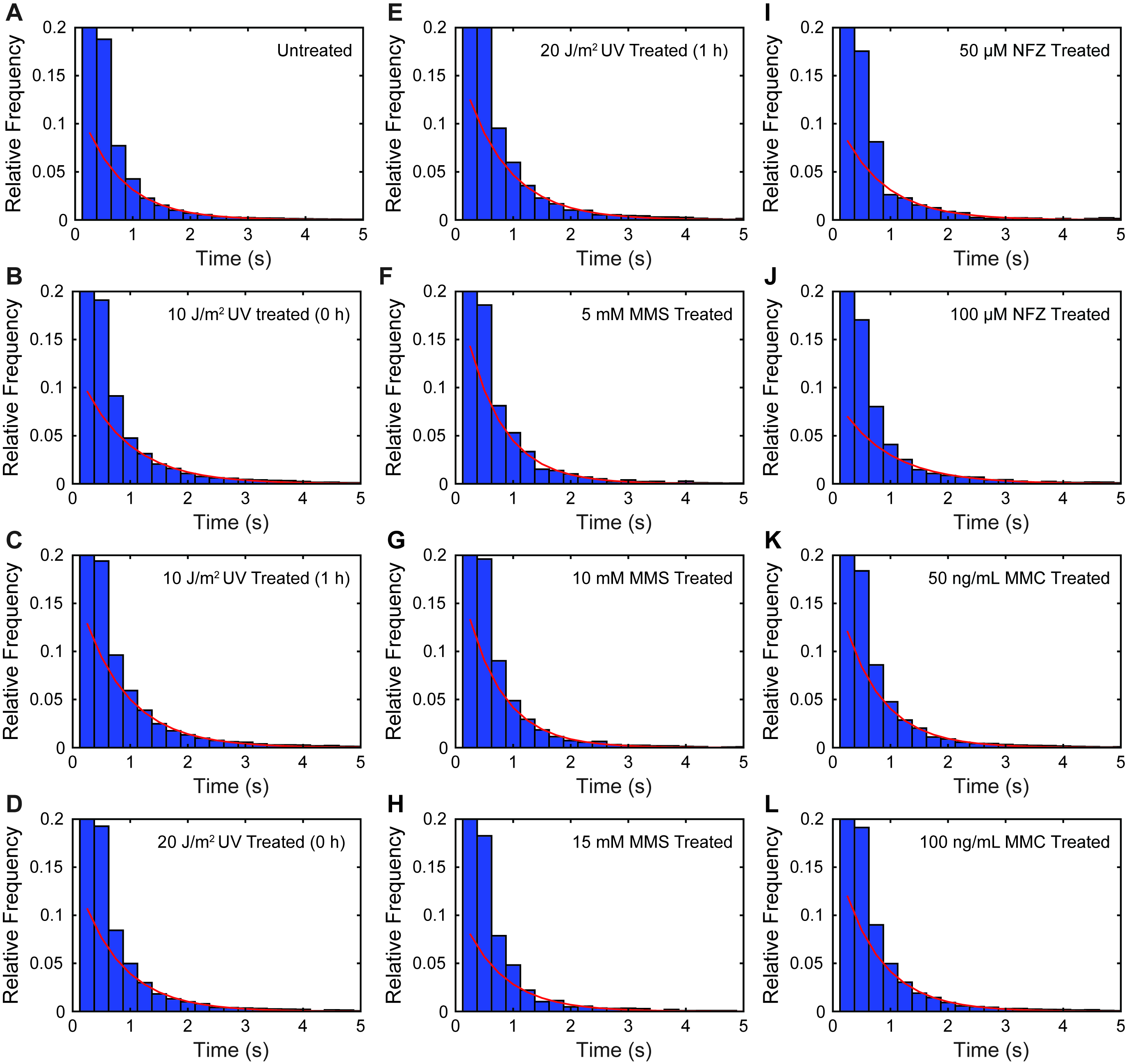

Supplement: S6 Fig — Distributions of apparent binding lifetime and corresponding exponential fits for (A) untreated cells and after treatment with (B) 10 J/m2 254 nm UV light (t = 0 h), (C) 10 J/m2 254 nm UV light (t = 1 h), (D) 20 J/m2 254 nm UV light (t = 0 h), (E) 20 J/m2 254 nm UV light (t = 1 h), (F) 5 mM MMS, (G) 10 mM MMS, (H) 15 mM MMS, (I) 100 µM NFZ, (J) 100 µM NFZ, (K) 50 ng/mL MMC, and (L) 100 ng/mL MMC. (Note that the y-axes are truncated to show the longer timescale behavior more clearly.). (TIF) [file pgen.1012246.s006.tif]
