# Supplementary material for: The B. subtilis translesion polymerase Pol Y1 is not strongly recruited to sites of replication upon different types of DNA damage
Source: PLoS Genet. 2026 Jul 14;22(7):e1012246. doi: 10.1371/journal.pgen.1012246 (PMC13387608; doi:10.1371/journal.pgen.1012246)
Supplement: S1 Text — Supplementary Tables A – I and Supplementary Methods. (PDF) [file pgen.1012246.s007.pdf]

“The *B. subtilis* translesion polymerase Pol Y1 is not strongly recruited to sites of replication upon different types of DNA damage”

Sophia R. Martinez-Whitman[1&], Chloe M. Santana[1&], Alyssa P. Campbell[1], Denholm T. Feldman[1], Isaac E.Z. Jabaley[1], Luke G. O’Neal[1], McKayla E. Marrin[1], Elizabeth S. Thrall[1]*
[1]Department of Chemistry and Biochemistry, Fordham University, Bronx, NY 10458
*****Correspondence: ethrall@fordham.edu
[&]Equal contributions

SUPPORTING INFORMATION

This PDF file includes:

Tables A to I
Supplementary Methods
Supplementary References

Supplementary Tables

Table A. Mutagenesis for different strains under different treatment conditions measured by the proportion of cells resistant to rifampicin (Rif^R) (mean ± standard deviation). All MMS, NFZ, and MMC incubations were performed for 1 h. A separate untreated dataset was collected in matched experiments for each treatment condition. (Note: * indicates statistically significant difference at the $p < 0.05$ level relative to WT for same treatment condition. ‡ indicates statistically significant difference at the $p < 0.05$ level for treated vs. untreated condition for the same strain.)

**Strain**	**WT**		**ΔPol Y1**		**ΔPol Y2**		**ΔPol Y1 ΔPol Y2**	
**Condition**	**Untreated**	**40 J/m^2 UV**	**Untreated**	**40 J/m^2 UV**	**Untreated**	**40 J/m^2 UV**	**Untreated**	**40 J/m^2 UV**
**Rif^R (per 10^8)**	0.9 ± 0.6	16 ± 4 *‡	0.7 ± 0.2	5 ± 3 *‡	0.6 ± 0.3	5 ± 1 *‡	0.7 ± 0.4	0.6 ± 0.6 *
**Condition**	**Untreated**	**10 mM MMS**	**Untreated**	**10 mM MMS**	**Untreated**	**10 mM MMS**	**Untreated**	**10 mM MMS**
**Rif^R (per 10^8)**	1.5 ± 0.8	11 ± 2 ‡	2 ± 1	9 ± 5 ‡	0.8 ± 0.6	10 ± 5 ‡	1 ± 1	8 ± 3 ‡
**Condition**	**Untreated**	**100 µM NFZ**	**Untreated**	**100 µM NFZ**	**Untreated**	**100 µM NFZ**	**Untreated**	**100 µM NFZ**
**Rif^R (per 10^8)**	1.5 ± 0.3	3.2 ± 0.6 ‡	1.9 ± 0.6	4 ± 1 ‡	1.4 ± 0.6	3 ± 2	1.8 ± 0.7	3 ± 1
**Condition**	**Untreated**	**200 ng/mL MMC**	**Untreated**	**200 ng/mL MMC**	**Untreated**	**200 ng/mL MMC**	**Untreated**	**200 ng/mL MMC**
**Rif^R (per 10^8)**	0.8 ± 0.6	12 ± 5 ‡	0.8 ± 0.5	14 ± 7 ‡	1.5 ± 0.9	2.1 ± 0.6 *	1.3 ± 0.5	2 ± 1 *

Table B. Cell length and width and number of DnaX foci per cell (mean ± standard error of the mean (S.E.M.)) under different treatment conditions. (Note: ‡ indicates difference was not statistically significant at the $p < 0.05$ level relative to the untreated condition.)

**Strain**	**Cell Length (µm)**	**Cell Width (µm)**	**Number of Foci per Cell**
Untreated	3.40 ± 0.02	0.699 ± 0.002	1.76 ± 0.02
10 J/m^2 UV (0 h)	3.40 ± 0.03 ‡	0.680 ± 0.002	1.67 ± 0.03
10 J/m^2 UV (1 h)	4.07 ± 0.04	0.698 ± 0.002 ‡	2.13 ± 0.04
20 J/m^2 UV (0 h)	3.64 ± 0.05	0.708 ± 0.004 ‡	1.70 ± 0.05 ‡
20 J/m^2 UV (1 h)	4.10 ± 0.04	0.692 ± 0.003 ‡	2.13 ± 0.05
5 mM MMS (1 h)	3.19 ± 0.03	0.711 ± 0.002	1.74 ± 0.04 ‡
10 mM MMS (1 h)	3.15 ± 0.04	0.664 ± 0.002	1.41 ± 0.04
15 mM MMS (1 h)	3.14 ± 0.03	0.677 ± 0.002	1.50 ± 0.02
50 µM NFZ (1 h)	2.95 ± 0.04	0.621 ± 0.003	1.46 ± 0.04
100 µM NFZ (1 h)	2.72 ± 0.02	0.616 ± 0.002	1.31 ± 0.02
50 ng/mL MMC (1 h)	3.99 ± 0.03	0.672 ± 0.002	1.83 ± 0.03 ‡
100 ng/mL MMC (1 h)	4.07 ± 0.04	0.681 ± 0.003	1.74 ± 0.04 ‡

Table C. Fold change in number of colony forming units per mL (CFUs/mL) for imaging cultures after different treatments (mean ± standard deviation).

**Condition**	**Untreated**	**DMF (1 h)**	**DMSO (1 h)**	
**Fold Change in CFUs/mL**	2.1 ± 0.4	1.9 ± 0.3	1.9 ± 0.2	
**Condition**	**10 J/m^2 UV (0 h)**	**10 J/m^2 UV (1 h)**	**20 J/m^2 UV (0 h)**	**20 J/m^2 UV (1 h)**
**Fold Change in CFUs/mL**	0.72 ± 0.08	1.3 ± 0.2	0.4 ± 0.1	0.9 ± 0.2
**Condition**	**5 mM MMS (1 h)**	**10 mM MMS (1 h)**	**15 mM MMS (1 h)**	
**Fold Change in CFUs/mL**	1.4 ± 0.3	0.9 ± 0.1	0.30 ± 0.03	
**Condition**	**50 µM NFZ (1 h)**	**100 µM NFZ (1 h)**	**250 µM NFZ (1 h)**	
**Fold Change in CFUs/mL**	1.1 ± 0.3	1.0 ± 0.1	1.2 ± 0.2	
**Condition**	**50 ng/mL MMC (1 h)**	**100 ng/mL MMC (1 h)**		
**Fold Change in CFUs/mL**	0.8 ± 0.2	0.5 ± 0.1		

Table D. Value of the mean radial distribution function $g(r)$ for Pol Y1-DnaX colocalization at the second smallest value of r (generally the maximum of the $g(r)$ curve) and the standard deviation at that r value for the 100 calculated $g(r)$ curves.

**Figure(s)**	**Condition**	**$g(r)$ ± Std. Dev.**
5A, 5B, 5C, 5D	Untreated	2.00 ± 0.07
5A	10 J/m^2 UV (0 h)	2.21 ± 0.09
	10 J/m^2 UV (1 h)	2.37 ± 0.09
	20 J/m^2 UV (0 h)	2.6 ± 0.2
	20 J/m^2 UV (1 h)	2.7 ± 0.1
5B	5 mM MMS (1 h)	2.0 ± 0.2
	10 mM MMS (1 h)	2.7 ± 0.3
	15 mM MMS (1 h)	2.4 ± 0.3
5C	50 µM NFZ (1 h)	4.5 ± 0.8
	100 µM NFZ (1 h)	2.6 ± 0.3
5D	50 ng/mL MMC (1 h)	2.8 ± 0.2
	100 ng/mL MMC (1 h)	2.7 ± 0.2

Table E. Pol Y1-Halo diffusion coefficient distribution fit parameters from MSD analysis (± uncertainties from 95% fit confidence intervals).

**Condition**	***D_1* (μm²/s)**	***A_1***	***D_2* (μm²/s)**	***A_2***	***D_3* (μm²/s)**	***A_3***
Untreated	0.082 ± 0.004	0.317 ± 0.032	1.0761 ± 0.086	0.477 ± 0.028	0.230 ± 0.036	0.206 ± 0.060
10 J/m² UV (0 h)	0.077 ± 0.005	0.299 ± 0.033	1.065 ± 0.087	0.473 ± 0.028	0.214 ± 0.031	0.228 ± 0.061
10 J/m² UV (1 h)	0.078 ± 0.083	0.324 ± 0.034	1.025 ± 0.095	0.439 ± 0.030	0.221 ± 0.032	0.240 ± 0.065
20 J/m² UV (0 h)	0.079 ± 0.005	0.343 ± 0.039	1.065 ± 0.099	0.426 ± 0.029	0.209 ± 0.032	0.231 ± 0.067
20 J/m² UV (1 h)	0.077 ± 0.005	0.336 ± 0.045	0.972 ± 0.098	0.376 ± 0.027	0.190 ± 0.025	0.289 ± 0.075
5 mM MMS (1 h)	0.081 ± 0.004	0.288 ± 0.025	1.134 ± 0.07	0.531 ± 0.023	0.230 ± 0.033	0.181 ± 0.048
10 mM MMS (1 h)	0.079 ± 0.004	0.327 ± 0.031	1.123 ± 0.10	0.468 ± 0.030	0.231 ± 0.037	0.206 ± 0.061
15 mM MMS (1 h)	0.073 ± 0.007	0.278 ± 0.052	1.051 ± 0.089	0.489 ± 0.027	0.180 ± 0.030	0.233 ± 0.080
50 μM NFZ (1 h)	0.070 ± 0.007	0.358 ± 0.062	1.002 ± 0.157	0.334 ± 0.035	0.179 ± 0.029	0.308 ± 0.097
100 μM NFZ (1 h)	0.074 ± 0.005	0.329 ± 0.038	0.959 ± 0.103	0.419 ± 0.034	0.212 ± 0.033	0.252 ± 0.072
50 ng/mL MMC (1 h)	0.076 ± 0.005	0.437 ± 0.056	1.005 ± 0.156	0.325 ± 0.035	0.195 ± 0.039	0.238 ± 0.092
100 ng/mL MMC (1 h)	0.075 ± 0.007	0.351 ± 0.064	0.931 ± 0.125	0.332 ± 0.032	0.180 ± 0.027	0.317 ± 0.096

Table F. Apparent and photobleaching-corrected Pol Y1-Halo binding lifetimes (± uncertainties from 95% fit confidence intervals). A photobleaching lifetime τ_{Bleach} = 1.10 ± 0.05 s measured previously(1) was used to correct apparent binding lifetimes for photobleaching.

**Condition**	τ_{app} **(s)**	τ_{bound} **(s)**
Untreated	0.70 ± 0.01	1.95 ± 0.04
10 J/m^2 UV (0 h)	0.84 ± 0.02	3 ± 1
10 J/m^2 UV (1 h)	0.79 ± 0.01	2.8 ± 0.8
20 J/m^2 UV (0 h)	0.75 ± 0.01	2.3 ± 0.6
20 J/m^2 UV (1 h)	0.76 ± 0.03	2.4 ± 0.7
5 mM MMS (1 h)	0.64 ± 0.03	1.5 ± 0.4
10 mM MMS (1 h)	0.64 ± 0.03	1.5 ± 0.4
15 mM MMS (1 h)	0.71 ± 0.05	2.0 ± 0.8
50 µM NFZ (1 h)	0.77 ± 0.04	2.5 ± 0.8
100 µM NFZ (1 h)	0.88 ± 0.04	4 ± 2
50 ng/mL MMC (1 h)	0.68 ± 0.02	1.8 ± 0.4
100 ng/mL MMC (1 h)	0.70 ± 0.02	2.0 ± 0.5

Table G. Oligonucleotides used in this study. (Lowercase letters indicate bases that do not prime on the template but provide homology for Gibson assembly of PCR fragments.)

**Number**	**Designation**	**Sequence (5′-3′)**
oEST028	yqjH-downstream-rev	CGACAACTTCAGATGGGCTGGTGTTC
oEST037	yqjH-upstream-for	CATCAGTCACCGTATTGACT
oEST038	yqjH-Nter-rev	TCGGCTCTTTCCCGGCATAA
oEST039	yqjH-Nter-spec-iso-for	ttatgccgggaaagagccgaGGATCCTTCTGCTCCCTCGC
oEST040	yqjH-Cter-spec-iso-rev	attcagcttttcttttcatcGACCAGGGAGCACTGGTCAA
oEST041	yqjH-Cter-for	GATGAAAAGAAAAGCTGAATCGC

Table H. *B. subtilis* bacterial strains used in this study.

Number	Designation or description	Relevant genotype	Construction or source strain designation	Reference
EST003 (WT)	*B. subtilis* prototrophic wild-type strain	PY79	—	(2, 3)
EST0081	PolC-Dendra2	PY79 *polC-dendra2 loxP-spec-loxP*	Gift of Xindan Wang (Indiana University); strain BWX2913	—
EST111	ΔPol Y1	PY79 *yqjH::loxP-spec-loxP*	EST111	(1)
EST117	ΔPol Y2	PY79 *yqjW::loxP-spec-loxP*	EST117	(1)
EST137	ΔPol Y1	PY79 *yqjH::loxP-kan-loxP*	Transformation: *yqjH::loxP-kan-loxP* → EST003	This study
EST143	Pol Y1-Halo	PY79 *yqjH-halo loxP-spec-loxP*	EST143	(1)
EST169	ΔPol Y1 ΔPol Y2	PY79 *yqjH::loxP-kan-loxP yqjW::loxP-spec-loxP*	Transformation: EST117 → EST137	This study
EST197	Pol Y1-Halo DnaX-mYPet	PY79 *yqjH-halo loxP-spec-loxP dnaX-mYpet cat Ω pWX340a*	EST197	(1)
EST371	P_{yneA}-GFP	NCIB 3610 *amyE*::P_{yneA}*-gfp-mut2 cat*	Gift of Yunrong Chai (Northeastern University); strain YCN036 (KG32)	(4)
EST375	P_{yneA}-GFP	PY79 *amyE*::P_{yneA}*-gfp-mut2 cat*	Transformation: EST371 → EST003	This study

Table I. Imaging dataset size.

Dataset/Condition	Figure(s)	Number of Days	Number of Replicates	Number of Cells	Number of Tracks or Foci
DnaX cellular localization<br>Untreated	4B, S3A	6	8	1,881	3,316
Pol Y1 cellular localization<br>Untreated	4B, S3A	6	8	1,881	31,649
DnaX cellular localization<br>10 J/m^2 UV (0 h)	4C, S3B	4	4	909	1,520
Pol Y1 cellular localization<br>10 J/m^2 UV (0 h)	4C, S3B	4	4	909	18,925
DnaX cellular localization<br>10 J/m^2 UV (1 h)	4D, S3C	4	4	858	1,828
Pol Y1 cellular localization<br>10 J/m^2 UV (1 h)	4D, S3C	4	4	858	27,667
DnaX cellular localization<br>20 J/m^2 UV (0 h)	S3D, S4A	3	3	296	501
Pol Y1 cellular localization<br>20 J/m^2 UV (0 h)	S3D, S4A	3	3	296	7,519
DnaX cellular localization<br>20 J/m^2 UV (1 h)	S3E, S4B	3	3	445	946
Pol Y1 cellular localization<br>20 J/m^2 UV (1 h)	S3E, S4B	3	3	445	14,993
DnaX cellular localization<br>5 mM MMS (1 h)	4E, S3F	3	3	594	1,034
Pol Y1 cellular localization<br>5 mM MMS (1 h)	4E, S3F	3	3	594	7,023
DnaX cellular localization<br>10 mM MMS (1 h)	S3G, S4C	3	3	488	686
Pol Y1 cellular localization<br>10 mM MMS (1 h)	S3G, S4C	3	3	488	4,108
DnaX cellular localization<br>15 mM MMS (1 h)	S3H, S4D	4	4	945	1,410
Pol Y1 cellular localization<br>15 mM MMS (1 h)	S3H, S4D	4	4	945	4,900
DnaX cellular localization<br>50 µM NFZ (1 h)	4F, S3I	4	4	579	846
Pol Y1 cellular localization<br>50 µM NFZ (1 h)	4F, S3I	4	4	579	1,565
DnaX cellular localization<br>100 µM NFZ (1 h)	S3J, S4E	5	5	1,638	2,148
Pol Y1 cellular localization<br>100 µM NFZ (1 h)	S3J, S4E	5	5	1,638	3,379
DnaX cellular localization<br>50 ng/mL MMC (1 h)	4G, S3K	4	4	981	1,800
Pol Y1 cellular localization<br>50 ng/mL MMC (1 h)	4G, S3K	4	4	981	10,325
DnaX cellular localization<br>100 ng/mL MMC (1 h)	S3L, S4F	4	4	663	1,151
Pol Y1 cellular localization<br>100 ng/mL MMC (1 h)	S3L, S4F	4	4	663	8,235
Pol Y1-DnaX $g(r)$<br>Untreated	5A, 5B, 5C, 5D	6	8	1,881	30,606

Pol Y1-DnaX *g*(*r*)<br>10 J/m^2 UV (0 h)	5A	4	4	909	18,441
Pol Y1-DnaX *g*(*r*)<br>10 J/m^2 UV (1 h)	5A	4	4	858	27,173
Pol Y1-DnaX *g*(*r*)<br>20 J/m^2 UV (0 h)	5A	3	3	296	2,482
Pol Y1-DnaX *g*(*r*)<br>20 J/m^2 UV (1 h)	5A	3	3	445	5,864
Pol Y1-DnaX *g*(*r*)<br>5 mM MMS (1 h)	5B	3	3	594	1,614
Pol Y1-DnaX *g*(*r*)<br>10 mM MMS (1 h)	5B	3	3	488	1,878
Pol Y1-DnaX *g*(*r*)<br>15 mM MMS (1 h)	5B	4	4	945	4,722
Pol Y1-DnaX *g*(*r*)<br>50 µM NFZ (1 h)	5C	4	4	579	1,454
Pol Y1-DnaX *g*(*r*)<br>100 µM NFZ (1 h)	5C	5	5	1,638	3,033
Pol Y1-DnaX *g*(*r*)<br>50 ng/mL MMC (1 h)	5D	4	4	981	3,323
Pol Y1-DnaX *g*(*r*)<br>100 ng/mL MMC (1 h)	5D	4	4	663	1,913
Pol Y1 D^*<br>Untreated	6A	6	8	1,881	29,120
Pol Y1 D^*<br>10 J/m^2 UV (0 h)	6B	4	4	909	17,446
Pol Y1 D^*<br>10 J/m^2 UV (1 h)	6C	4	4	858	25,449
Pol Y1 D^*<br>20 J/m^2 UV (0 h)	S5A	3	3	296	6,881
Pol Y1 D^*<br>20 J/m^2 UV (1 h)	S5B	3	3	445	13,760
Pol Y1 D^*<br>5 mM MMS (1 h)	6D	3	3	594	6,462
Pol Y1 D^*<br>10 mM MMS (1 h)	S5C	3	3	488	3,729
Pol Y1 D^*<br>15 mM MMS (1 h)	S5D	4	4	945	4,512
Pol Y1 D^*<br>50 µM NFZ (1 h)	6E	4	4	579	1,390
Pol Y1 D^*<br>100 µM NFZ (1 h)	S5E	5	5	1,638	2,993
Pol Y1 D^*<br>50 ng/mL MMC (1 h)	6F	4	4	981	9,385
Pol Y1 D^*<br>100 ng/mL MMC (1 h)	S5F	4	4	663	7,381
P*yneA-gfp* Intensity<br>Untreated	S1	2	2	587	N/A
P*yneA-gfp* Intensity<br>40 J/m^2 UV (1 h)	S1	2	2	489	N/A
P*yneA-gfp* Intensity<br>10 mM MMS (1 h)	S1	2	2	713	N/A
P*yneA-gfp* Intensity<br>100 µM NFZ (1 h)	S1	2	2	647	N/A
P*yneA-gfp* Intensity	S1	2	2	364	N/A

200 ng/mL MMC (1 h)					
Pol Y1 binding lifetime Untreated	S6A	5	8	1,586	31,640
Pol Y1 binding lifetime 10 J/m^2 UV (0 h)	S6B	4	4	699	16,162
Pol Y1 binding lifetime 10 J/m^2 UV (1 h)	S6C	3	4	577	20,885
Pol Y1 binding lifetime 20 J/m^2 UV (0 h)	S6D	3	3	345	8,941
Pol Y1 binding lifetime 20 J/m^2 UV (1 h)	S6E	3	3	299	14,448
Pol Y1 binding lifetime 5 mM MMS (1 h)	S6F	3	3	406	4,903
Pol Y1 binding lifetime 10 mM MMS (1 h)	S6G	3	3	466	5,166
Pol Y1 binding lifetime 15 mM MMS (1 h)	S6H	4	4	743	4,599
Pol Y1 binding lifetime 50 µM NFZ (1 h)	S6I	4	4	481	2,860
Pol Y1 binding lifetime 100 µM NFZ (1 h)	S6J	5	5	1,359	7,216
Pol Y1 binding lifetime 50 ng/mL MMC (1 h)	S6K	4	4	692	14,396
Pol Y1 binding lifetime 100 ng/mL MMC (1 h)	S6L	4	4	555	11,957

Supplementary Methods

Overview of strain construction strategy:

Introduction of chromosomal genetic modifications by transformation of genomic DNA or double-stranded DNA (dsDNA) fragments assembled via Gibson assembly(5) was performed as described previously.(1) All oligonucleotides and bacterial strains used in this study are listed in Tables G and H, respectively. Construction details for all new strains are summarized below.

Detailed strain construction information:

EST137: ΔPol Y1. The *yqjH* upstream was amplified from strain EST003 using oligonucleotides oEST037 and oEST038. The spec-loxP-spec cassette was amplified from strain EST081 using oligonucleotides oEST039 and oEST040. The *yqjH* downstream was amplified from strain EST003 using oligonucleotides oEST041 and oEST028. The three fragments were joined by Gibson assembly and transformed into strain EST003.

EST169: ΔPol Y1 ΔPol Y2. The *yqjW::loxP-spec-loxP* allele was transferred from strain EST117 to strain EST137 by transformation with genomic DNA.

EST375: P_{yneA}-GFP. The *amyE::*P_{yneA}*-gfp-mut2 cat* allele was transferred from strain EST371 to strain EST003 by transformation with genomic DNA.

Supplementary References

1. Marrin,M.E., Foster,M.R., Santana,C.M., Choi,Y., Jassal,A.S., Rancic,S.J., Greenwald,C.R., Drucker,M.N., Feldman,D.T. and Thrall,E.S. (2024) The translesion polymerase Pol Y1 is a constitutive component of the *B. subtilis* replication machinery. *Nucleic Acids Research*, **52**, 9613–9629.

2. Zeigler,D.R., Prágai,Z., Rodriguez,S., Chevreux,B., Muffler,A., Albert,T., Bai,R., Wyss,M. and Perkins,J.B. (2008) The Origins of 168, W23, and Other *Bacillus subtilis* Legacy Strains. *J Bacteriol*, **190**, 6983–6995.

3. Schroeder,J.W. and Simmons,L.A. (2013) Complete Genome Sequence of *Bacillus subtilis* Strain PY79. *Genome Announc*, **1**, e01085-13.

4. Gozzi,K., Ching,C., Paruthiyil,S., Zhao,Y., Godoy-Carter,V. and Chai,Y. (2017) Bacillus subtilis utilizes the DNA damage response to manage multicellular development. *npj Biofilms Microbiomes*, **3**, 8.

5. Gibson,D.G., Young,L., Chuang,R.-Y., Venter,J.C., Hutchison,C.A. and Smith,H.O. (2009) Enzymatic assembly of DNA molecules up to several hundred kilobases. *Nat Methods*, **6**, 343–345.
