# Supplementary material for: The B. subtilis translesion polymerase Pol Y1 is not strongly recruited to sites of replication upon different types of DNA damage
Source: PLoS Genet. 2026 Jul 14;22(7):e1012246. doi: 10.1371/journal.pgen.1012246 (PMC13387608; doi:10.1371/journal.pgen.1012246)
Supplement: Attachment — Submitted filename: PGENETICS-D-26-00322_Feedback.pdf [file pgen.1012246.s008.pdf]

“The *B. subtilis* translesion polymerase Pol Y1 is not strongly recruited to sites of replication upon different types of DNA damage.” From the group of Dr. Thrall.

Comments to the Authors:

Martinez-Whitman et al. examine how induced DNA damage affects the location and movement of Y-family DNA polymerase Pol Y1 in the Gram-positive bacterium *Bacillus subtilis,* while investigating the role of Y-family DNA polymerases Pol Y1 and Pol Y2 in damage tolerance and induced mutagenesis. This investigation extends a previous significant finding from the Thrall lab (PMID: 39051562), in which Pol Y1 was proposed to be constitutively associated with the replisome even in the absence of induced DNA damage, unlike the archetype DinB in E. coli. In the current study, the authors used four different DNA-damaging agents to test the tolerance of the Pol Y1 deletion and compared it with that of the Pol Y2 mutant. The authors show that Pol Y1 is important for tolerance to UV-induced DNA damage and mutagenesis, while Pol Y2 is dispensable, reaffirming previous studies. Surprisingly, the Pol Y1 deletion mutant was modestly or negligibly sensitive to three other DNA-damaging agents (MMS, NFZ, and MMC) and showed no discernible effect on induced mutagenesis. Pol Y2 showed similar, varying effects. Using single-molecule tracking, the authors show that Pol Y1 is not prominently enriched at replication sites post exposure to four DNA damage inducers, despite the variable inducer-specific differences, strongly supporting their previous study. Overall, the study establishes that Pol Y1 association with the replisome is largely independent of induced DNA damage, unlike the well-characterized E. coli DinB, thereby highlighting the diversity of TLS DNA polymerases across bacteria. The manuscript is well written, and the findings are of significant interest, which can be further strengthened with the points discussed below:

1. Statistical analysis of single-molecule data is largely ignored. The diffusion kinetics of Pol Y1 upon exposure to induced DNA damage must be evaluated for statistical significance by comparing with the untreated condition.

This is important and may be necessary to conclude strongly: Page 14, line 359-362 – “Thus, although changes in diffusion were larger than changes in Pol Y1 enrichment at replication forks, the results were not consistent across different types of DNA damage, and even the largest effects were not substantial”.

The authors can substantiate their claims with appropriate statistical measures, since the observed changes could be significant, especially given the variable effects of the absence of Pol Y1 on survival and its potential to induce mutagenesis post-treatment in some cases. Importantly, it will reinforce the study’s major claim that Pol Y1 is not specifically enriched following induced DNA damage.

2. Statistical validation to choose between the 2-state or 3-state model of Pol Y1 can be presented in the current study.

3. Statistical support is required for the survival data presented in Figure 1.

4. The author may consider nuancing the statement in Page 12, line 315-317, “Pol Y1 is not strongly recruited”. In light of the author's previous work on the constitutive association of Pol Y1, it is possible that Pol Y1 diffusion dynamics are altered by the type of induced DNA damage and the resulting response, rather than by active recruitment. Also, in the discussion, the results from the current study do not seem to be highlighted in the context of the constant association of Pol Y1 with the replisome.

5. As described in the methods, it seems that the mutation frequency to rifampicin resistance was enumerated; however, throughout the manuscript, it is described as a rate. The authors may want to describe it as mutation frequency rather than rate, as this accurately reflects the experiment performed, particularly when the sample size is limited to 5 cultures.

6. The study and findings are significant and thorough; however, the manuscript title does not completely reflect them. Is Pol Y1 not strongly recruited or enriched upon induced DNA damage? The authors may want to comment or review the title.

Minor Points:

1. The concentration of Rifampicin used is missing.
2. Page 4, line 60 – regulation instead of “regular”?
3. Page 7, line 140 –the authors meant to say Pol Y1?
4. Page 9, line 215 – DnaX foci formation is marked in the bottom panel (as described in Fig. 3 legend)
5. Page 9, line 217 – Pol Y1 molecule is marked in the middle panel (as described in Fig. 3 legend)
6. In Figure 4 and S2, it would be easier to compare and comprehend if all high concentrations are placed in one panel (maybe the main figure) and the rest in another place. Only NFZ at a lower concentration is displayed in the main figure.
