# Supplementary material for: The B. subtilis translesion polymerase Pol Y1 is not strongly recruited to sites of replication upon different types of DNA damage
Source: PLoS Genet. 2026 Jul 14;22(7):e1012246. doi: 10.1371/journal.pgen.1012246 (PMC13387608; doi:10.1371/journal.pgen.1012246)
Supplement: Attachment — Submitted filename: Response to Reviewers.pdf [file pgen.1012246.s010.pdf]

We thank the reviewers for their constructive and detailed feedback on our work. Below, we give responses to each reviewer critique. In addition to numerous changes to the text and figures to clarify points of confusion or address reviewer comments, we have added three new substantial experiments:

1. Measurement of Pol Y1 binding lifetime to confirm that there are no substantial changes upon DNA damage
2. Imaging of an SOS reporter strain to confirm that the tested damaging agents induce SOS
3. Validation that the introduction of the Pol Y1-HaloTag fusion does not confer a survival defect upon UV damage

In addition, we have substantially revised the Discussion section in response to feedback from the reviewers.

We have also corrected Figure 2 after identifying two small errors in the plotted data, but these errors have no impact on the qualitative interpretation of the results.

Our comments to reviewers are in blue and new manuscript text is in red.

Reviewer #1:

In this work, Martinez-Whitman investigate the requirement of B. subtilis translesion polymerases PolY1 and PolY2 under different kinds of DNA damage. TLS has been well-studied in E. coli, but as the authors state, TLS polymerases vary across bacterial species and the requirement/ mechanism of these specialized polymerases has been less investigated. B. subtilis encodes for a PolIV-family TLS polymerase (PolY1) and PolV-family TLS polymerase (PolY2, with key differences in its mode of activation that distinguish it from UmuDC). Here the authors assess the essentiality, recruitment and mutagenic role of these polymerases in vegetatively growing B. subtilis exposed to NFZ, MMS, UV and MMC damage. Each of these damaging agents induce a distinct type of DNA modification, and have shown differential requirements of PolIV and PolV in E. coli. The authors find that survival in the absence of PolY1 is more affected UV, NFZ, and MMS conditions to varying degrees. In case of mutagenesis, PolY1 deletion clearly affects rates of rif resistance development in UV conditions, while PolY2 is required for MMC-induced mutagenesis. Finally, the authors track the recruitment of PolY1 to the replisome under damage, and find that PolY1 recruitment does not change under damage. However, diffusion rates are differentially affected. Overall, the authors provide a comprehensive picture of PolY1 and PolY2 requirement in B. subtilis under DNA damaging conditions, and highlight differences between TLS function in E. coli and B. subtilis.

This is a well-executed study, that has important implications on our understanding of DNA damage tolerance mechanisms beyond E. coli. The authors are correct to point to the gap in

studies on this pathway outside of E. coli and their findings make a compelling case for such studies to be extended further. I am highly supportive of this work, and have a few suggestions/ comments for the authors to consider prior to publication:

1. Imaging of PolY1:
a. Can the authors measure the dwell time of the PolY1 molecules? Although the level of colocalization does not change, it is possible that molecules within the localization have altered dynamics. Along with providing the apparent diffusion coefficient measurements, authors could also provide dwell time distributions.

We have added binding lifetime analysis for new data collected with a longer integration time (250 ms), because the short integration time (13.9 ms) used in other experiments does not allow for reliable binding lifetime measurements. These new data are presented in Figure S6 and Table S6. In brief, we find that there are very minimal changes in binding lifetime upon DNA damage, which only reach statistical significance in one case. We thank the reviewer for suggesting this experiment, which provides further confirmation that Pol Y1 dynamics change minimally upon DNA damage.

We have added a description of the binding lifetime experiments and analysis to the relevant Methods sections on p. 23. We have added a description of the findings in the Results section on p. 15 – 16:

> Although diffusion analysis did not reveal substantial changes in Pol Y1 mobility upon DNA damage, we also characterized its binding lifetime under the different treatment conditions as an alternative measurement of its dynamics. In brief, we used a longer 250 ms integration time to visualize statically bound Pol Y1 molecules selectively and measured the duration of each trajectory. Because either Pol Y1 dissociation or JFX_{554} photobleaching could cause a trajectory to end, we corrected this apparent binding lifetime (τ_{app}) by the dye photobleaching lifetime measured in fixed cells. We then fit the resulting distribution of corrected binding lifetimes to a single-exponential decay to determine the mean binding lifetime (τ_{bound}) (Figure S6 and Table S6). Consistent with our prior study, we found that $\tau_{bound} \approx 2$ s in untreated cells (1.95 ± 0.04 s vs. 1.9 ± 0.4 s previously). Previously, we showed that 4-NQO treatment had a minimal impact on the Pol Y1 binding lifetime. Likewise, none of the treatment conditions tested in this study led to a greater than approximately two-fold change in τ_{bound}, and the average change was smaller (Table S6). The 95% fit confidence intervals on τ_{bound} for all treatments overlapped with those in untreated cells, with one exception (10 J/m^2 UV treatment, $t = 0$ h time point) indicating that the measured differences in binding lifetime may not be statistically significant. Thus, the binding lifetime analysis is consistent with the diffusion analysis in revealing minimal changes in Pol Y1 mobility upon DNA damage.

b. Does the intensity of DnaX or PolY1 in a localization change under damage? Stoichiometry measurements can additionally strengthen the conclusion that PolY1 recruitment appears to not change overall across a range of DNA damaging agents.

We use a low concentration of the JFX_{554} dye for labeling of Pol Y1-Halo molecules in order to obtain the sparse labeling conditions needed for reliable single-particle tracking. Therefore, we do not observe static Pol Y1 "foci" comprised of multiple molecules; instead, each Pol Y1 spot represents a single molecule, and the spot intensities do not provide information about the copy number of Pol Y1 in a complex. Therefore, we do not believe it is possible to measure Pol Y1 stoichiometry using our current dataset.

In the case of the DnaX-mYPet foci, the intensity of the foci (in principle) provides information about the stoichiometry. However, in previous studies, we have found that intensity measurements using near-TIRF excitation are somewhat variable day-to-day. Although we have not found this variability to affect localization or tracking analysis, it will of course affect measured intensity values. In experiments where we wish to look for potentially small changes in fluorescence intensities, we image samples back-to-back on the same day, which we were not able to do for the large set of damaging conditions in this study. Given these concerns, we would prefer not to include an analysis of DnaX focus intensity, although the analysis could be reported with these caveats if the reviewer felt that it was essential.

c. Authors should include survival comparison of untagged and tagged PolY1 under the different types of damaging agents used in the present study.

The functionality of the Pol Y1-Halo fusion used in these experiments was tested in a prior study (Marrin, et al. *Nucleic Acids Res.* **2024**, *16*, 9613). The fusion strain was found to have comparable resistance to the drug 4-NQO as the wild-type (WT) strain; in contrast, a Pol Y1 deletion strain was sensitized to 4-NQO treatment. We agree with the reviewer that additional characterization of this fusion strain would be valuable. However, given the lack of sensitization observed for the Pol Y1 deletion strain upon MMC or NFZ treatment, we believe that assaying survival of the Pol Y1-Halo fusion strain upon treatment with these drugs would not be informative. Likewise, although the Pol Y1 deletion strain is sensitized to MMS treatment, the effect is relatively small, and thus the dynamic range for detecting impairment of the Pol Y1-Halo fusion is quite limited.

Therefore, we have characterized the survival of this fusion strain upon UV treatment, the condition in this study under which we observed the greatest impact of Pol Y1 deletion. Consistent with our prior findings for 4-NQO treatment, we find that the cells bearing the Pol

Y1-Halo fusion (with or without the DnaX-mYPet fusion) are not sensitized to UV damage relative to WT, further validating the use of this fusion for microscopy.

These data are shown in new Figure S2 and described in the main text on p. 10:

> As additional confirmation that the HaloTag does not affect Pol Y1 function, we assayed the survival of fusion strains upon UV treatment. We found that the Pol Y1-Halo fusion had no effect on cell survival upon UV treatment relative to WT, either in the presence or absence of the DnaX-mYPet fusion (Figure S2), validating its use in microscopy.

d. It is not fully clear to me how the authors interpret the significant enrichment of PolY1 under NFZ treatment (Fig. 5). Authors should also comment on the observed lack of relation between enrichment, change in binding kinetics, mutagenesis and survival in case of PolY1.

We do not have a clear interpretation of the increase in colocalization observed for the 50 µM NFZ treatment condition and therefore we did not suggest an interpretation because we did not want to speculate too much. However, we agree that it should be addressed and have added a line to the Discussion section on p. 18 stating that we do not have a clear interpretation, especially in light of the fact that this increase is not observed for the higher NFZ concentration. We also address the point that we do not observe changes in localization or mobility of Pol Y1 even for drugs to which it promotes survival:

> Given this model from *E. coli*, it is surprising that we do not observe changes in Pol Y1 dynamics or enrichment at replication sites upon DNA damage, particularly for damaging agents (UV and MMS in this study, and 4-NQO in our previous study(11)) to which it promotes survival. The only exception was NFZ treatment, for which we observed a moderate increase in colocalization for the 50 µM concentration, but not for the higher 100 µM concentration, making interpretation difficult.

e. Imaging and mutagenesis experiments are carried out at different doses of DNA damage (MMC and UV), and hence are not fully comparable. Authors should clearly explain the choice of these doses for the two types of experiments, and can acknowledge the caveat that the doses are not matched for direct comparison of recruitment and mutagenic outcome.

The reviewer is correct about this discrepancy. In the case of UV and MMC, we chose to perform mutagenesis experiments using doses that had been investigated in previous studies, either of *B. subtilis* Pol Y1 and Pol Y2 specifically (for UV) or with *B. subtilis* in general (for MMC). However, cell survival after treatment with these doses was low enough that we were concerned that the majority of cells analyzed microscopically would be non-viable, leading to results that might not be physiological; therefore, we chose to use a lower dose for imaging. In

both cases, the doses used in mutagenesis assays differ by only a factor of two from doses used in imaging experiments, so we felt that the discrepancy was not too significant. However, we agree that this choice should be stated clearly and explained in the manuscript, and we have modified the main text on p. 11 and 12 to do so:

> We note that these UV doses are lower than the 40 J/m^2 dose used in our mutagenesis assays. Although this higher dose was selected to match prior studies of Pol Y1 and Pol Y2 mutagenesis, we chose not to use it in imaging experiments due to the significant loss of viability observed at the lower 20 J/m^2 dose, raising the concern that imaging results might not be physiologically relevant if a substantial majority of cells were non-viable.
>
> Given the significant reduction in viability at the highest concentration, we decided to use only the lower two concentrations in imaging experiments, even though we used the higher concentration in mutagenesis assays to match prior studies in *B. subtilis*. As for UV treatment, we cannot exclude the possibility that this modest differences in MMC dose leads to qualitative differences in Pol Y1 behavior, although we believe that it is unlikely.

2. SOS induction: Please provide evidence for SOS induction in these damaging agents, as PolY2 expression is under SOS control.

To address this point, we have imaged a previously characterized SOS reporter construct in which GFP expression is under the control of the promoter of *yneA*, an SOS-regulated gene that inhibits cell division upon DNA damage (Gozzi, et al. *npj Biofilms Microbiomes*. **2017**, *3*, 8). Because our study includes a number of different DNA damaging conditions, we chose to test the treatments used in the mutagenesis assays. In matched experiments on the same day, we imaged untreated cells and cells treated with UV, MMS, NFZ, and MMC. We found that all four treatments induced SOS, with a higher degree of induction for UV and MMC and a lower degree of induction for MMS and NFZ.

These results are reported in Figure S1 and in the main text on p. 9:

> Pol Y2 is present at very low copy number in the absence of DNA damage, but it is upregulated upon induction of the SOS response. Therefore, to test whether the treatments used in these mutagenesis assays induce SOS, and thus Pol Y2 expression, we imaged cells bearing a previously characterized SOS reporter construct. In this transcriptional P_{yneA}-*gfp* reporter, GFP expression is under the control of the promoter of *yneA*, an SOS-regulated gene that inhibits cell division upon DNA damage. As expected, we observed low basal fluorescence in normally growing cells, but a strong increase upon DNA damage. These results are consistent with prior reports that treatment with UV,

MMC, and the alkylating agent ethyl methanesulfonate (EMS) all induce SOS in *B. subtilis* and that NFZ and MMS treatment induce SOS in *E. coli*. To quantify the degree of SOS induction, we calculated the normalized cellular fluorescence by measuring the total fluorescence intensity for each cell and dividing by the cell area (Figure S1). The average normalized cellular fluorescence increased relative to the untreated condition by a factor of 3.4-fold upon UV treatment, 1.6-fold upon MMS treatment, 2.1-fold upon NFZ treatment, and 3.3-fold upon MMC treatment ($p < 0.05$ in all cases; fold-changes in median intensities were almost identical). Thus, all four treatment conditions induce SOS, with UV and MMC leading to greater induction than NFZ and MMS. Taken together, these results suggest that the lack of contribution of Pol Y2 to survival for all four damaging agents and to mutagenesis for MMS and NFZ does not simply reflect a lack of expression; for example, 40 J/m^2 UV treatment strongly induces SOS, yet Pol Y2 has no impact on cell survival under this condition (Figure 1A).

We have updated the Methods section on p. 21 – 23 to describe the experiment and analysis. We have also added experimental details to the SI, including details on the introduction of the SOS reporter construct into our PY79 WT background.

3. Discussion:
a. There is a lot of information to unpack in this paper. The authors could include a summary table of the various effects (or absence of the same) of PolY1/ PolY2 under the different types of DNA damage observed in this study, comparing to what is known for E. coli PolIV and PolV.

We thank the reviewer for this helpful suggestion. We have added Table 1 to the main text to summarize our survival and mutagenesis results and to compare them to prior literature findings for the *E. coli* TLS polymerases.

b. The discussion lacks perspective on how these cells may be dealing with the lesions in the presence or absence of TLS polymerases. For example, do the authors think nucleotide excision repair handles most of these lesions, with replication in the gap being carried out by the replicative polymerase or PolI? Indeed, in B. subtilis, PolI has been shown to play a role along with TLS polymerases. It is also possible that TLS is non-mutagenic in some context, and cost of mutations balance out TLS action in other cases. Authors could comment on how they think lesion tolerance or repair occurs in B. subtilis.

We thank the reviewer for raising this question, which we agree is an important one. We have revised the Discussion section substantially in response to this feedback and related feedback from Reviewer #2. Please see our response to the Reviewer #2 comments for greater detail on the changes that we have made.

4. Minor comments:
a. Could the authors comment on why PolY2 was not imaged. If it is an issue with the tagging of this polymerase, a line in the manuscript stating the same should be included.

The reviewer is correct that we have not yet succeeded in creating a functional Pol Y2 fusion, possibly due to its position as the first gene in an operon. We also felt that this manuscript already contained a substantial amount of data, and that future careful microscopic characterization of Pol Y2 should represent its own study. We have added a sentence to the Discussion on p. 19 providing this information:

> We have found that construction of a functional Pol Y2 fusion for microscopy is complicated by its position as the first gene in an operon, but further research should investigate its behavior at the single-molecule level.

b. It is understandable that survival differs significantly across the various types of damage. However, the y-axis reporting survival in Fig. 1 should kept constant (log or linear) to enable comparison across the various types of damage.

The reviewer is correct that survival is much lower for the UV and MMS treatments shown in Figure 1 than for the NFZ and MMC treatments. The original figure already used a log scale for the *y*-axis, but the axis range was different for UV/MMS and NFZ/MMC. Replotting the NFZ and MMC data using the same range as UV/MMS would make it difficult to see the survival results for these damaging agents. However, to avoid confusion, we have added a note to the figure caption:

> Note that all *y*-axes are on a log scale.

c. For statistical tests described in table S1, were any corrections applied to account for multiple comparisons.

We did not apply corrections for multiple comparisons because we were testing a small set of well-motivated biological hypotheses and did not want to risk introducing Type 2 errors (false negatives). However, to ensure that our conclusions are statistically robust, we have now implemented the Benjamini-Hochberg procedure to correct for multiple comparisons. Applying this correction to the mutagenesis data in Table S1 leads to no change in the statistically significant comparisons. (We note that even the more stringent Bonferroni correction would not produce a change.)

We have now applied this procedure to all the significance testing in the manuscript and have revised the Methods section on p. 24 to describe it:

The Benjamini-Hochberg procedure, implemented in the MATLAB function mafdr, was used to correct for multiple comparisons between mutant strains and the WT strain (for survival and mutagenesis assays) or between untreated and treated cells (for imaging data). Corrected p-values were assessed for significance at the $p < 0.05$ level.

d. Table S5 appears to be mis-labeled.

We thank the reviewer for pointing out this error. We have corrected the table caption to refer to Pol Y1-Halo.

e. Maintain nomenclature when refering to B. subtilis PolY1 and PolY2. It is confusing to the reader if the authors sometimes refer to PolY1/ Y2 as PolIV or PolV.

We agree with the reviewer that the *B. subtilis* TLS polymerases should be referred to as Pol Y1/Pol Y2 exclusively. We have carefully checked the manuscript and corrected any erroneous references to Pol Y1/Pol Y2 as Pol IV/Pol V. All remaining mentions of Pol IV/Pol V in the text are references to the *E. coli* TLS polymerases.

Reviewer #2:

The manuscript measures the activity of the translesion polymerases Pol Y1 and Pol Y2 in Bacillus subtilis after treatment with multiple DNA damaging agents. Work on translesion polymerases in bacteria has been focused on Escherichia coli, where the orthologues of Pol Y1 and Pol Y2 (Pol IV and Pol V) have been extensively characterized. Importantly, E. coli translesion polymerases have a preference for repair of particular types of damage, correlating with their binding kinetics as reported by single-molecule microscopy. A previous publication by their group showed no clear changes in the binding kinetics of Pol Y1 after treatment with 4-NQO, despite a contribution to cell survival. Here, the authors extend this work to other DNA damaging agents by first, determining the contribution of Pol Y1 and Pol Y2 to cell survival and mutagenesis, and then by characterizing the localization, co-localization to the replication fork, and DNA binding of Pol IV using fluorescence microscopy. Their results show clear differences with the usage of B. subitlis translesion polymerases when compared to E. coli, showing only small contributions to survival and mutagenesis, and minimal (or uncorrelated with survival) changes in the binding kinetics of Pol Y1 after treatment.

Previous work on translesion polymerases in B. subtilis was done using different growth conditions, complicating its interpretation. This paper reports a systematic study of the role of translesion polymerases to survival of multiple DNA damaging agents, correcting this limitation.

Furthermore, this work consolidates the suggestion that, differently from E. coli, there are no clear changes in the localization or binding kinetics of Pol Y1 that correlate to repair activity. In this sense, the work opens multiple questions to be addressed in the future.

My main criticism of the paper is that it lacks a clear interpretation and discussion of the implications of the data. For example, the authors describe the contributions of Pol Y1 and Pol Y2 to multiple DNA damaging agents. Using deletion mutants for these genes, the authors observe only marginal viability loss and mutagenesis changes for most of the treatments. This suggests that translesion polymerases do not play a major role in the survival of B. subtilis. This is not only different to E. coli, but probably different to most other organisms (take as example, the classical genetic description of UV and translesion polymerases in yeast doi: 10.1093/genetics/68.1.21). The authors should provide information on the impact to survival and mutagenesis in other organisms for context. But if it is indeed something unique about B. subtilis, how can this be explained? Is the replicative polymerase PolC able to bypass DNA damage? Can DnaE contribute to it? Is there redundancy coded somewhere else? Similarly, the imaging results can be discussed at a greater extent. For example, what is the relevance in the localization changes in DnaX after treatment? 10 J/m^2 has only a small effect on survival, but the localization is clearly different. Are there issues with DNA replication? The patterns are similar to those observed for other treatments where survival was much lower. Is the number of foci per cell changing? Can this influence the co-localization results? In isolation, these result do not provide much biological meaning.

The authors could also make better use of the discussion section. In the current manuscript there is plenty of overlap with what was already stated in the results section. The authors should avoid redundancy and instead write about their work in context to previous literature.

We thank the reviewer for this constructive feedback. Although we feel that our work raises many questions that we are not in a position to answer at this point, we agree that we should attempt to contextualize our findings better. We have revised the Discussion section substantially to address these comments:

- We have condensed the summary of results substantially, only highlighting key findings, and we refer to the new Table 1 instead.
- We have reviewed the literature on the role of TLS in contributing to DNA damage survival in *E. coli* and have added a new paragraph comparing and contrasting the importance of this pathway in the two species. We have discussed possibilities for alternative enzymes that may contribute to DNA damage tolerance in *B. subtilis*, including the replicative polymerase DnaE and the repair polymerase Pol I.
- We have summarized the literature on how different types of DNA lesions are repaired in *E. coli* and *B. subtilis* and discussed the evidence that Pol Y1 and Pol Y2 may have a role in transcription-coupled nucleotide excision repair.

- We have added discussion of the localization changes that we observe for the DnaX foci to make our interpretation of these results clearer.
- We have made the point that because Pol Y1 is always enriched near sites of replication, even in the absence of induced damage, it may not require further enrichment for its action in TLS, in contrast to its *E. coli* homolog Pol IV.

We hope that the new Discussion section does a better job of contextualizing our findings and highlighting open questions that will require future studies to address.

With respect to the question about colocalization, radial distribution function analysis should not be affected by changes in the number of DnaX foci per cell (or by changes in cell morphology). In this procedure, the experimental distribution of Pol Y1-DnaX distances is normalized by a random distribution of distances that is calculated using the actual cell outlines and the actual location and number of DnaX foci in the experimental dataset. Thus, changes in cell morphology or DnaX foci due to DNA damage will also change the random distribution, preventing any artifacts in the resulting colocalization analysis. We have added a sentence to p. 13 of the Results section to clarify this point:

> Because this analysis normalizes the experimental distribution of Pol Y1-DnaX distances by a random distribution calculated for each cell in the dataset, it is unaffected by changes in cell morphology or in the number or localization of DnaX foci.

Other points:
- In figure 4, the diagram of the short axis position (bottom right, Fig 4A) is confusion, particularly when the rest of the figure has plots that are paired. I initially interpreted them as long and short axis, but they represent DnaX and Pol Y1.

We thank the reviewer for identifying this point of confusion. We have revised Figure 4A to reposition the short axis cartoon as shown below, which we hope will facilitate interpretation.

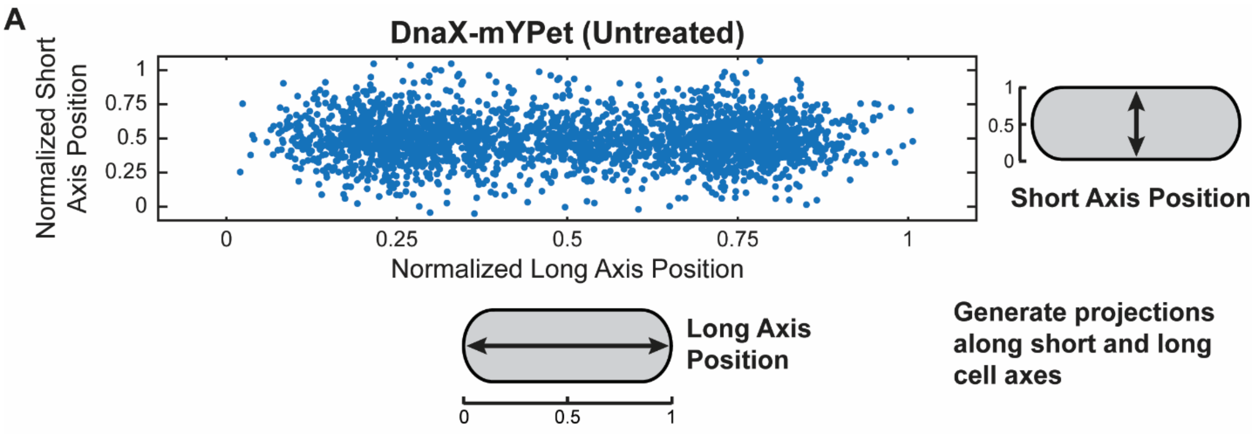

We have also added the following line to the Figure 4 and Figure S4 captions to indicate that the short axis localization data are shown in a separate figure:

> The corresponding short axis projections are shown in Figure S3.

- In figure 4, do the projection of localizations include bound and diffusive molecules? Should be stated in the figure legend.

The Pol Y1 localizations in Figure 4 represent all detected trajectories, not just bound molecules. As in our prior study, very short trajectories were excluded from all analyses due to the inability to calculate reliable apparent different coefficient values for these trajectories, as well as the higher likelihood that they represent false positives. We have revised the Methods section on p. 23 and the Figure 4 caption to make this clear, also adding information about the colocalization and diffusion analyses:

> Methods: Localization and colocalization analyses were performed for all trajectories with at least five localizations and diffusion analysis was performed for all trajectories with at least four steps.
>
> Figure 4 caption: Long axis projections of DnaX foci (left) and all detected Pol Y1 trajectories…

- In figure 5, what are the imaging conditions used for Pol Y1-Halo? Does this represent bound molecules in single molecule microscopy? The conditions used should be clearly stated in the results, figure legend and/ or the methods section

We thank the reviewer for identifying this point of confusion. All imaging in the original manuscript was performed using a short exposure time (13.9 ms) to allow visualization of both static and mobile Pol Y1 molecules. Thus, the colocalization analysis in Figure 5 includes all detected trajectories (subject to the trajectory length conditions described in the previous response). As suggested by Reviewer #1, we have now added binding lifetime analysis, which was performed using a longer 250 ms exposure time. We have updated the Methods section on p. 22 to describe the different exposure times. We have also updated the Figure 5 and 6 captions to make clear that it represents analysis of all detected Pol Y1 trajectories:

> Methods: For localization, colocalization, and diffusion analyses, movies were recorded using a short integration time (13.9 ms) to enable detection of both mobile and static Pol Y1 molecules. For binding lifetime analysis, a longer integration time (250 ms) was used for selective detection of static molecules.

Figure 5 caption: Pol Y1-DnaX $g(r)$ for all detected trajectories in untreated cells…

Figure 6 caption: Pol Y1 D^* distributions for all detected trajectories in (A) untreated cells…

Reviewer #3:

Martinez-Whitman et al. examine how induced DNA damage affects the location and movement of Y-family DNA polymerase Pol Y1 in the Gram-positive bacterium *Bacillus subtilis,* while investigating the role of Y-family DNA polymerases Pol Y1 and Pol Y2 in damage tolerance and induced mutagenesis. This investigation extends a previous significant finding from the Thrall lab (PMID: 39051562), in which Pol Y1 was proposed to be constitutively associated with the replisome even in the absence of induced DNA damage, unlike the archetype DinB in E. coli. In the current study, the authors used four different DNA-damaging agents to test the tolerance of the Pol Y1 deletion and compared it with that of the Pol Y2 mutant. The authors show that Pol Y1 is important for tolerance to UV-induced DNA damage and mutagenesis, while Pol Y2 is dispensable, reaffirming previous studies. Surprisingly, the Pol Y1 deletion mutant was modestly or negligibly sensitive to three other DNA-damaging agents (MMS, NFZ, and MMC) and showed no discernible effect on induced mutagenesis. Pol Y2 showed similar, varying effects. Using single-molecule tracking, the authors show that Pol Y1 is not prominently enriched at replication sites post exposure to four DNA damage inducers, despite the variable inducer-specific differences, strongly supporting their previous study. Overall, the study establishes that Pol Y1 association with the replisome is largely independent of induced DNA damage, unlike the well-characterized E. coli DinB, thereby highlighting the diversity of TLS DNA polymerases across bacteria. The manuscript is well written, and the findings are of significant interest, which can be further strengthened with the points discussed below:

1. Statistical analysis of single-molecule data is largely ignored. The diffusion kinetics of Pol Y1 upon exposure to induced DNA damage must be evaluated for statistical significance by comparing with the untreated condition.
This is important and may be necessary to conclude strongly: Page 14, line 359-362 – “Thus, although changes in diffusion were larger than changes in Pol Y1 enrichment at replication forks, the results were not consistent across different types of DNA damage, and even the largest effects were not substantial”.
The authors can substantiate their claims with appropriate statistical measures, since the observed changes could be significant, especially given the variable effects of the absence of Pol Y1 on survival and its potential to induce mutagenesis post-treatment in some cases. Importantly,

it will reinforce the study's major claim that Pol Y1 is not specifically enriched following induced DNA damage.

We are not aware of a standard statistical approach for determining significance for this type of diffusion population analysis. However, as a way to test whether differences are likely to be meaningful, we calculate uncertainties on the static population percentages based on the 95% fit confidence intervals (Table S5). If the confidence intervals for a given fit parameter overlap for two samples, then we consider the difference between them unlikely to be meaningful. In the first version of the manuscript, we did not explicitly refer to these confidence intervals, but we agree that a discussion should be included. We have now significantly revised our discussion of the diffusion results on p. 15 to refer to these confidence intervals and have focused the discussion on the few differences that appear significant:

> Next, we asked whether treatment with DNA damaging agents altered the mobility of Pol Y1 molecules in the cell. Although there were changes in the relative populations of Pol Y1 upon DNA damage, most of these changes were relatively small (Figures 6B – F and S4A – F, Table S5). With only a few exceptions, the 95% fit confidence intervals for each population in damaged cells overlapped the corresponding intervals for each population in undamaged cells, suggesting that these small changes are not statistically significant. In particular, changes for both UV treatments were minimal, with the only statistically significant change being an approximately 20% decrease in the mobile population at the t = 1 h timepoint for the 20 J/m^2 dosage. MMS treatment likewise had only minor effects on Pol Y1 mobility, with only a small (~ 10%) increase in the mobile population for the 5 mM concentration appearing distinguishable from the untreated condition. NFZ treatment produced larger changes in Pol Y1 mobility than either UV or MMS, but only a decrease of approximately 30% in the mobile population for the 50 μM concentration appeared significant. MMC led to the largest changes in Pol Y1 diffusion, but without an obvious dose-dependent effect. The lower concentration (50 ng/mL) produced a larger increase in the static population (~ 40%), paired with a corresponding decrease in the mobile population (~ 30%), both of which were distinguishable from the untreated condition. The higher concentration (100 ng/mL) led to a similar decrease in the mobile population, which did reach statistical significance, but with a smaller (~ 10%) increase in the static population, which did not. Thus, DNA damage led to relatively modest changes in Pol Y1 mobility in the cell, with many of the measured changes not proving statistically significant.

We have likewise incorporated this type of confidence interval analysis into our discussion of the new binding lifetime data on p. 15 – 16. As for the diffusion data, this analysis confirms that almost all of the binding lifetime changes upon DNA damage do not reach statistical significance.

With respect to the radial distribution function colocalization analysis, we now report the standard deviation of the 100 calculated $g(r)$ curves at the maximum value in Table S4, which provides a measure of variability due to the finite sample size. Many of the measured changes in $g(r)$ values fall outside the range of one standard deviation, although our interpretation is that they are relatively modest changes in comparison to the several-fold changes observed previously for *E. coli* Pol IV.

2. Statistical validation to choose between the 2-state or 3-state model of Pol Y1 can be presented in the current study.

As we reported in our previous study (Marrin, et al. *Nucleic Acids Res.* **2024**, *16*, 9613), the 2-state model does not fit the Pol Y1 diffusion distributions well visually. We have confirmed that the same is true qualitatively for our current dataset. Below is Figure S6J from that article, demonstrating the failure of a 2-state fit to describe the data:

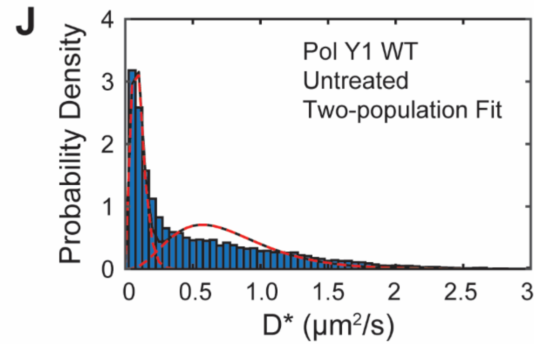

As a more quantitative statistical measure, we have now compared the Akaike Information Criterion (AIC) values for the 2-state vs. 3-state fits. The Akaike Information Criterion (AIC) value reflects the goodness of fit of a model while also penalizing model complexity, allowing for comparison between two models with different numbers of parameters. A smaller AIC value indicates a better fit; when AIC values are negative, a more negative value indicates a better fit. For the untreated cell diffusion data shown in Figure 6A of this manuscript, the AIC value is -52.7 for the 2-state fit and -127.8 for the 3-state fit. The smaller (more negative) AIC value for the 3-state fit indicates that, consistent with visual inspection, it is a better model for the diffusion data. We have calculated the AIC values for all diffusion datasets and confirmed that in all cases the 3-state fit is better.

We have updated the Methods section on p. 23 to provide further explanation for the choice of a 3-state model:

The D^* distributions were fit to a 3-state model, with a static DNA-bound population, a highly mobile population, and a population with intermediate mobility, which may reflect molecules binding transiently to DNA. We note that visual comparison of an alternative 2-state model fit indicated that it could not describe the data well, as reported in our prior study.(11) To validate this choice statistically, we compared the Akaike Information Criterion (AIC) values for 2-state vs. 3-state fits; this metric reflects goodness of fit while penalizing model complexity. Smaller AIC values indicate a better fit; when the AIC values are negative, more negative values indicate a better fit. For all diffusion datasets, the AIC values for the 3-state model were smaller than those for the 2-state model, confirming that the 3-state model better describes the data. As an example, for the untreated cell diffusion data shown in Figure 6A, the AIC value is -52.7 for the 2-state fit and -127.8 for the 3-state fit.

We have also updated the Results section on p. 14 to give a possible physical interpretation for the different diffusive populations:

The resulting fit yields a static DNA-bound population with low mobility ($D^* \approx 0.08$ $\mu m^2/s$), a highly mobile population ($D^* \approx 1.0$ $\mu m^2/s$), and an intermediate population ($D^* \approx 0.2$ $\mu m^2/s$); this population may represent molecules interacting transiently with DNA.

3. Statistical support is required for the survival data presented in Figure 1.

We are not aware of a procedure to test for significance globally across an entire survival curve. Therefore, we have chosen to compare the survival rates separately for each treatment dose, using the same approach described in our response to reviewer #1 to correct for the fact that we are making multiple comparisons to the WT strain. We have modified the discussion of the survival assays in the Results section on p. 6 – 7 to indicate significant differences at the $p < 0.05$ level. We have also removed a comment about the possibility of reduced survival for the ΔPol Y1 strain at the highest NFZ concentration tested because the difference did not meet the significance threshold.

We have modified the Methods section on p. 23 to describe this new procedure:

For significance testing of survival datasets, the survival rate for each mutant strain was compared to that of the WT strain at each individual treatment condition (*i.e.*, at each UV dose or drug concentration).

4. The author may consider nuancing the statement in Page 12, line 315-317, "Pol Y1 is not strongly recruited". In light of the author's previous work on the constitutive association of Pol Y1, it is possible that Pol Y1 diffusion dynamics are altered by the type of induced DNA damage

and the resulting response, rather than by active recruitment. Also, in the discussion, the results from the current study do not seem to be highlighted in the context of the constant association of Pol Y1 with the replisome.

We agree that this language could be clearer. In summarizing the Pol Y1-DnaX colocalization results, we were trying to make the point that we did not observe a substantial increase in colocalization upon DNA damage, in contrast to prior findings for *E. coli*. But the reviewer is correct that our prior work showed (and data in this study confirm) that Pol Y1 is constitutively enriched near sites of replication during normal growth, and so it is possible that Pol Y1 activity upon DNA damage does not require further enrichment or active recruitment. We have modified this sentence to read:

> Taken together, these results show that there is no substantial increase in Pol Y1 enrichment at replication sites upon DNA damage, in contrast to prior findings for *E. coli* Pol IV.

Further, we have revised the discussion to make this point; please see our response to review #2 for more details.

5. As described in the methods, it seems that the mutation frequency to rifampicin resistance was enumerated; however, throughout the manuscript, it is described as a rate. The authors may want to describe it as mutation frequency rather than rate, as this accurately reflects the experiment performed, particularly when the sample size is limited to 5 cultures.

We thank the reviewer for this suggestion and have corrected the language throughout the main text and SI to refer to the "mutation frequency" instead of the "mutation rate."

6. The study and findings are significant and thorough; however, the manuscript title does not completely reflect them. Is Pol Y1 not strongly recruited or enriched upon induced DNA damage? The authors may want to comment or review the title.

The title was our attempt to convey the big-picture conclusion of the manuscript: that DNA damage does not induce substantial changes in the dynamics of Pol Y1 or in its colocalization with sites of DNA replication, in contrast to prior observations for its *E. coli* homolog Pol IV. We have used the word "recruited" to convey that Pol Y1 is not further enriched at replication sites upon DNA damage. Although we would be open to a different title, we have been unable to think of one that conveys this main idea in a clearer and more concise way.

Minor Points:
1. The concentration of Rifampicin used is missing.

We thank the reviewer for identifying this omission. We have revised the Methods section on p. 21 to include the rifampicin concentration used in these assays:

> For all assays, the overnight cultures were serially diluted and plated on LB Lennox agar plates with or without 10 µg/mL Rif.

2. Page 4, line 60 – regulation instead of "regular"?
3. Page 7, line 140 –the authors meant to say Pol Y1?
4. Page 9, line 215 – DnaX foci formation is marked in the bottom panel (as described in Fig. 3 legend)
5. Page 9, line 217 – Pol Y1 molecule is marked in the middle panel (as described in Fig. 3 legend)

We thank the reviewer for identifying these typos and have corrected them.

6. In Figure 4 and S2, it would be easier to compare and comprehend if all high concentrations are placed in one panel (maybe the main figure) and the rest in another place. Only NFZ at a lower concentration is displayed in the main figure.

We agree that this change makes sense for consistency. We have updated Figures 4 and 6 to show the 50 µM NFZ data and have updated what are now Figures S4 and S5 to show the 100 µM NFZ data.
