# Supplementary material for: The B. subtilis translesion polymerase Pol Y1 is not strongly recruited to sites of replication upon different types of DNA damage
Source: PLoS Genet. 2026 Jul 14;22(7):e1012246. doi: 10.1371/journal.pgen.1012246 (PMC13387608; doi:10.1371/journal.pgen.1012246)
Supplement: Peer review history

# Peer review: Decision Letter 0

**DOI:** 10.1371/journal.pgen.1012246.r001

**Authors:** Danielle Garsin, Jue Wang

27 Apr 2026

PGENETICS-D-26-00322

The *B. subtilis*  translesion polymerase Pol Y1 is not strongly recruited to sites of replication upon different types of DNA damage

PLOS Genetics

Dear Dr. Thrall,

Thank you for submitting your manuscript to PLOS Genetics. After careful consideration, we feel that it has merit but does not fully meet PLOS Genetics's publication criteria as it currently stands. Therefore, we invite you to submit a revised version of the manuscript that addresses the points raised during the review process.

Please submit your revised manuscript by Jun 26 2026 11:59PM. If you will need more time than this to complete your revisions, please reply to this message or contact the journal office at plosgenetics@plos.org. When you're ready to submit your revision, log on to https://www.editorialmanager.com/pgenetics/ and select the 'Submissions Needing Revision' folder to locate your manuscript file.

Please include the following items when submitting your revised manuscript:

* A letter that responds to each point raised by the editor and reviewer(s). You should upload this letter as a separate file labeled 'Response to Reviewers'. This file does not need to include responses to any formatting updates and technical items listed in the 'Journal Requirements' section below.

* A marked-up copy of your manuscript that highlights changes made to the original version. You should upload this as a separate file labeled 'Revised Manuscript with Track Changes'.

* An unmarked version of your revised paper without tracked changes. You should upload this as a separate file labeled 'Manuscript'.

If you would like to make changes to your financial disclosure, competing interests statement, or data availability statement, please make these updates within the submission form at the time of resubmission. Guidelines for resubmitting your figure files are available below the reviewer comments at the end of this letter.

We look forward to receiving your revised manuscript.

Kind regards,

Jue D. Wang

Academic Editor

PLOS Genetics

Danielle Garsin

Section Editor

PLOS Genetics

Aimée Dudley

Editor-in-Chief

PLOS Genetics

Anne Goriely

Editor-in-Chief

PLOS Genetics

**Journal Requirements:**

If the reviewer comments include a recommendation to cite specific previously published works, please review and evaluate these publications to determine whether they are relevant and should be cited. There is no requirement to cite these works unless the editor has indicated otherwise.

1) We ask that a manuscript source file is provided at Revision. Please upload your manuscript file as a .doc, .docx, .rtf or .tex. If you are providing a .tex file, please upload it under the item type u2018LaTeX Source Fileu2019 and leave your .pdf version as the item type u2018Manuscriptu2019.

2) We noticed that you used the phrase 'data not shown' in the manuscript. We do not allow these references, as the PLOS data access policy requires that all data be either published with the manuscript or made available in a publicly accessible database. Please amend the supplementary material to include the referenced data or remove the references.

3) Please upload all main figures as separate Figure files in .tif or .eps format. For more information about how to convert and format your figure files please see our guidelines:

https://journals.plos.org/plosgenetics/s/figures

4) We have noticed that you have uploaded Supporting Information files, but you have not included a list of legends. Please add a full list of legends for your Supporting Information files after the references list.

5) Please  detailed Financial Disclosure statement. This is published with the article. It must therefore be completed in full sentences and contain the exact wording you wish to be published.

If you did not receive any funding for this study, please simply state: u201cThe authors received no specific funding for this work.u201d

**Reviewers' comments:**

Reviewer's Responses to Questions

**Comments to the Authors:**

**Please note here if the review is uploaded as an attachment.**

Reviewer #1: In this work, Martinez-Whitman investigate the requirement of B. subtilis translesion polymerases PolY1 and PolY2 under different kinds of DNA damage. TLS has been well-studied in E. coli, but as the authors state, TLS polymerases vary across bacterial species and the requirement/ mechanism of these specialized polymerases has been less investigated. B. subtilis encodes for a PolIV-family TLS polymerase (PolY1) and PolV-family TLS polymerase (PolY2, with key differences in its mode of activation that distinguish it from UmuDC). Here the authors assess the essentiality, recruitment and mutagenic role of these polymerases in vegetatively growing B. subtilis exposed to NFZ, MMS, UV and MMC damage. Each of these damaging agents induce a distinct type of DNA modification, and have shown differential requirements of PolIV and PolV in E. coli. The authors find that survival in the absence of PolY1 is more affected UV, NFZ, and MMS conditions to varying degrees. In case of mutagenesis, PolY1 deletion clearly affects rates of rif resistance development in UV conditions, while PolY2 is required for MMC-induced mutagenesis. Finally, the authors track the recruitment of PolY1 to the replisome under damage, and find that PolY1 recruitment does not change under damage. However, diffusion rates are differentially affected. Overall, the authors provide a comprehensive picture of PolY1 and PolY2 requirement in B. subtilis under DNA damaging conditions, and highlight differences between TLS function in E. coli and B. subtilis.

This is a well-executed study, that has important implications on our understanding of DNA damage tolerance mechanisms beyond E. coli. The authors are correct to point to the gap in studies on this pathway outside of E. coli and their findings make a compelling case for such studies to be extended further. I am highly supportive of this work, and have a few suggestions/ comments for the authors to consider prior to publication:

1. Imaging of PolY1:

a. Can the authors measure the dwell time of the PolY1 molecules? Although the level of colocalization does not change, it is possible that molecules within the localization have altered dynamics. Along with providing the apparent diffusion coefficient measurements, authors could also provide dwell time distributions.

b. Does the intensity of DnaX or PolY1 in a localization change under damage? Stoichiometry measurements can additionally strengthen the conclusion that PolY1 recruitment appears to not change overall across a range of DNA damaging agents.

c. Authors should include survival comparison of untagged and tagged PolY1 under the different types of damaging agents used in the present study.

d. It is not fully clear to me how the authors interpret the significant enrichment of PolY1 under NFZ treatment (Fig. 5). Authors should also comment on the observed lack of relation between enrichment, change in binding kinetics, mutagenesis and survival in case of PolY1.

e. Imaging and mutagenesis experiments are carried out at different doses of DNA damage (MMC and UV), and hence are not fully comparable. Authors should clearly explain the choice of these doses for the two types of experiments, and can acknowledge the caveat that the doses are not matched for direct comparison of recruitment and mutagenic outcome.

2. SOS induction: Please provide evidence for SOS induction in these damaging agents, as PolY2 expression is under SOS control.

3. Discussion:

a. There is a lot of information to unpack in this paper. The authors could include a summary table of the various effects (or absence of the same) of PolY1/ PolY2 under the different types of DNA damage observed in this study, comparing to what is known for E. coli PolIV and PolV.

b. The discussion lacks perspective on how these cells may be dealing with the lesions in the presence or absence of TLS polymerases. For example, do the authors think nucleotide excision repair handles most of these lesions, with replication in the gap being carried out by the replicative polymerase or PolI? Indeed, in B. subtilis, PolI has been shown to play a role along with TLS polymerases. It is also possible that TLS is non-mutagenic in some context, and cost of mutations balance out TLS action in other cases. Authors could comment on how they think lesion tolerance or repair occurs in B. subtilis.

4. Minor comments:

a. Could the authors comment on why PolY2 was not imaged. If it is an issue with the tagging of this polymerase, a line in the manuscript stating the same should be included.

b. It is understandable that survival differs significantly across the various types of damage. However, the y-axis reporting survival in Fig. 1 should kept constant (log or linear) to enable comparison across the various types of damage.

c. For statistical tests described in table S1, were any corrections applied to account for multiple comparisons.

d. Table S5 appears to be mis-labeled.

e. Maintain nomenclature when refering to B. subtilis PolY1 and PolY2. It is confusing to the reader if the authors sometimes refer to PolY1/ Y2 as PolIV or PolV.

Reviewer #2: The manuscript measures the activity of the translesion polymerases Pol Y1 and Pol Y2 in Bacillus subtilis after treatment with multiple DNA damaging agents. Work on translesion polymerases in bacteria has been focused on Escherichia coli, where the orthologues of Pol Y1 and Pol Y2 (Pol IV and Pol V) have been extensively characterized. Importantly, E. coli translesion polymerases have a preference for repair of particular types of damage, correlating with their binding kinetics as reported by single-molecule microscopy. A previous publication by their group showed no clear changes in the binding kinetics of Pol Y1 after treatment with 4-NQO, despite a contribution to cell survival. Here, the authors extend this work to other DNA damaging agents by first, determining the contribution of Pol Y1 and Pol Y2 to cell survival and mutagenesis, and then by characterizing the localization, co-localization to the replication fork, and DNA binding of Pol IV using fluorescence microscopy. Their results show clear differences with the usage of B. subitlis translesion polymerases when compared to E. coli, showing only small contributions to survival and mutagenesis, and minimal (or uncorrelated with survival) changes in the binding kinetics of Pol Y1 after treatment.

Previous work on translesion polymerases in B. subtilis was done using different growth conditions, complicating its interpretation. This paper reports a systematic study of the role of translesion polymerases to survival of multiple DNA damaging agents, correcting this limitation. Furthermore, this work consolidates the suggestion that, differently from E. coli, there are no clear changes in the localization or binding kinetics of Pol Y1 that correlate to repair activity. In this sense, the work opens multiple questions to be addressed in the future.

My main criticism of the paper is that it lacks a clear interpretation and discussion of the implications of the data. For example, the authors describe the contributions of Pol Y1 and Pol Y2 to multiple DNA damaging agents. Using deletion mutants for these genes, the authors observe only marginal viability loss and mutagenesis changes for most of the treatments. This suggests that translesion polymerases do not play a major role in the survival of B. subtilis. This is not only different to E. coli, but probably different to most other organisms (take as example, the classical genetic description of UV and translesion polymerases in yeast doi: 10.1093/genetics/68.1.21). The authors should provide information on the impact to survival and mutagenesis in other organisms for context. But if it is indeed something unique about B. subtilis, how can this be explained? Is the replicative polymerase PolC able to bypass DNA damage? Can DnaE contribute to it? Is there redundancy coded somewhere else? Similarly, the imaging results can be discussed at a greater extent. For example, what is the relevance in the localization changes in DnaX after treatment? 10 J/m^2 has only a small effect on survival, but the localization is clearly different. Are there issues with DNA replication? The patterns are similar to those observed for other treatments where survival was much lower. Is the number of foci per cell changing? Can this influence the co-localization results? In isolation, these result do not provide much biological meaning.

The authors could also make better use of the discussion section. In the current manuscript there is plenty of overlap with what was already stated in the results section. The authors should avoid redundancy and instead write about their work in context to previous literature.

Other points:

- In figure 4, the diagram of the short axis position (bottom right, Fig 4A) is confusion, particularly when the rest of the figure has plots that are paired. I initially interpreted them as long and short axis, but they represent DnaX and Pol Y1.

- In figure 4, do the projection of localizations include bound and diffusive molecules? Should be stated in the figure legend.

- In figure 5, what are the imaging conditions used for Pol Y1-Halo? Does this represent bound molecules in single molecule microscopy? The conditions used should be clearly stated in the results, figure legend and/ or the methods section

Reviewer #3: Comments to the Authors - attached.

**********

**Have all data underlying the figures and results presented in the manuscript been provided?**

Large-scale datasets should be made available via a public repository as described in the *PLOS Genetics*  data availability policy, and numerical data that underlies graphs or summary statistics should be provided in spreadsheet form as supporting information.

Reviewer #1: Yes

Reviewer #2: Yes

Reviewer #3: Yes

**********

PLOS authors have the option to publish the peer review history of their article (what does this mean?). If published, this will include your full peer review and any attached files.

If you choose “no”, your identity will remain anonymous but your review may still be made public.

**Do you want your identity to be public for this peer review?**  For information about this choice, including consent withdrawal, please see our Privacy Policy.

Reviewer #1: No

Reviewer #2: No

Reviewer #3: No

[NOTE: If reviewer comments were submitted as an attachment file, they will be attached to this email and accessible via the submission site. Please log into your account, locate the manuscript record, and check for the action link "View Attachments". If this link does not appear, there are no attachment files.]

**Figure resubmission:**

While revising your submission, we strongly recommend that you use PLOS’s NAAS tool (https://ngplosjournals.pagemajik.ai/artanalysis) to test your figure files. NAAS can convert your figure files to the TIFF file type and meet basic requirements (such as print size, resolution), or provide you with a report on issues that do not meet our requirements and that NAAS cannot fix.

After uploading your figures to PLOS’s NAAS tool - https://ngplosjournals.pagemajik.ai/artanalysis, NAAS will process the files provided and display the results in the "Uploaded Files" section of the page as the processing is complete. If the uploaded figures meet our requirements (or NAAS is able to fix the files to meet our requirements), the figure will be marked as "fixed" above. If NAAS is unable to fix the files, a red "failed" label will appear above. When NAAS has confirmed that the figure files meet our requirements, please download the file via the download option, and include these NAAS processed figure files when submitting your revised manuscript.

**Reproducibility:**

To enhance the reproducibility of your results, we recommend that authors of applicable studies deposit laboratory protocols in protocols.io, where a protocol can be assigned its own identifier (DOI) such that it can be cited independently in the future. Additionally, PLOS ONE offers an option to publish peer-reviewed clinical study protocols. Read more information on sharing protocols at https://plos.org/protocols?utm_medium=editorial-email&utm_source=authorletters&utm_campaign=protocols

Attachment Submitted filename: PGENETICS-D-26-00322_Feedback.pdf

# Author comment: Author response to Decision Letter 1

**DOI:** 10.1371/journal.pgen.1012246.r002

18 Jun 2026

Attachment Submitted filename: Response to Reviewers.pdf

# Peer review: Decision Letter 1

**DOI:** 10.1371/journal.pgen.1012246.r003

**Authors:** Danielle Garsin, Jue Wang

7 Jul 2026

Dear Dr Thrall,

We are pleased to inform you that your manuscript entitled "The B. subtilis translesion polymerase Pol Y1 is not strongly recruited to sites of replication upon different types of DNA damage" has been editorially accepted for publication in PLOS Genetics. Congratulations!

Before your submission can be formally accepted and sent to production you will need to complete our formatting changes, which you will receive in a follow up email. Please be aware that it may take several days for you to receive this email; during this time no action is required by you. Please note: the accept date on your published article will reflect the date of this provisional acceptance, but your manuscript will not be scheduled for publication until the required changes have been made.

Once your paper is formally accepted, an uncorrected proof of your manuscript will be published online ahead of the final version, unless you’ve already opted out via the online submission form. If, for any reason, you do not want an earlier version of your manuscript published online or are unsure if you have already indicated as such, please let the journal staff know immediately at plosgenetics@plos.org.

In the meantime, please log into Editorial Manager at https://www.editorialmanager.com/pgenetics/, click the "Update My Information" link at the top of the page, and update your user information to ensure an efficient production and billing process. Note that PLOS requires an ORCID iD for all corresponding authors. Therefore, please ensure that you have an ORCID iD and that it is validated in Editorial Manager. To do this, go to ‘Update my Information’ (in the upper left-hand corner of the main menu), and click on the Fetch/Validate link next to the ORCID field.  This will take you to the ORCID site and allow you to create a new iD or authenticate a pre-existing iD in Editorial Manager.

If you have a press-related query, or would like to know about making your underlying data available (as you will be aware, this is required for publication), please see the end of this email. If your institution or institutions have a press office, please notify them about your upcoming article at this point, to enable them to help maximise its impact. Inform journal staff as soon as possible if you are preparing a press release for your article and need a publication date.

Thank you again for supporting open-access publishing; we are looking forward to publishing your work in PLOS Genetics!

Yours sincerely,

Jue D. Wang

Academic Editor

PLOS Genetics

Danielle Garsin

Section Editor

PLOS Genetics

Aimée Dudley

Editor-in-Chief

PLOS Genetics

Anne Goriely

Editor-in-Chief

PLOS Genetics

www.plosgenetics.org

BlueSky: @plos.bsky.social

----------------------------------------------------

Comments from the reviewers (if applicable):

Reviewer's Responses to Questions

**Comments to the Authors:**

**Please note here if the review is uploaded as an attachment.**

Reviewer #1: The authors have fully addressed my comments. I congratulate them on the excellent work.

Reviewer #2: The authors have responded to all my queries

Reviewer #3: This is a revision of an interesting manuscript that demonstrates the localization and kinetics of a highly conserved DNA polymerase, Pol Y1, which offers an alternate model for the activity of the DinB family of DNA polymerases.

The revised manuscript satisfactorily addressed all the questions and concerns. The revised manuscript presents an exciting work, and I might only request the authors to consider the following suggestion:

If the authors do not find a suitable statistical test to evaluate the diffusion kinetic data presented in Table S5, I would suggest rephrasing or excluding this sentence since overlapping 95% CI doesn’t always warrant the absence of a statistical difference:

“With only a few exceptions, the 95% fit confidence intervals for each population in damaged cells overlapped the corresponding intervals for each population in undamaged cells, suggesting that these small changes are not statistically significant”.

**********

**Have all data underlying the figures and results presented in the manuscript been provided?**

Large-scale datasets should be made available via a public repository as described in the *PLOS Genetics*  data availability policy, and numerical data that underlies graphs or summary statistics should be provided in spreadsheet form as supporting information.

Reviewer #1: None

Reviewer #2: Yes

Reviewer #3: Yes

**********

PLOS authors have the option to publish the peer review history of their article (what does this mean?). If published, this will include your full peer review and any attached files.

If you choose “no”, your identity will remain anonymous but your review may still be made public.

**Do you want your identity to be public for this peer review?**  For information about this choice, including consent withdrawal, please see our Privacy Policy.

Reviewer #1: No

Reviewer #2: No

Reviewer #3: No

----------------------------------------------------

**Data Deposition**

If you have submitted a Research Article or Front Matter that has associated data that are not suitable for deposition in a subject-specific public repository (such as GenBank or ArrayExpress), one way to make that data available is to deposit it in the Dryad Digital Repository. As you may recall, we ask all authors to agree to make data available; this is one way to achieve that. A full list of recommended repositories can be found on our website.

The following link will take you to the Dryad record for your article, so you won't have to re‐enter its bibliographic information, and can upload your files directly:

http://datadryad.org/submit?journalID=pgenetics&manu=PGENETICS-D-26-00322R1

More information about depositing data in Dryad is available at http://www.datadryad.org/depositing. If you experience any difficulties in submitting your data, please contact help@datadryad.org for support.

Additionally, please be aware that our data availability policy requires that all numerical data underlying display items are included with the submission, and you will need to provide this before we can formally accept your manuscript, if not already present.

----------------------------------------------------

**Press Queries**

If you or your institution will be preparing press materials for this manuscript, or if you need to know your paper's publication date for media purposes, please inform the journal staff as soon as possible so that your submission can be scheduled accordingly. Your manuscript will remain under a strict press embargo until the publication date and time. This means an early version of your manuscript will not be published ahead of your final version. PLOS Genetics may also choose to issue a press release for your article. If there's anything the journal should know or you'd like more information, please get in touch via plosgenetics@plos.org.

# Editor report: Acceptance letter

**DOI:** 10.1371/journal.pgen.1012246.r004

**Authors:** Danielle Garsin, Jue Wang

PGENETICS-D-26-00322R1

The B. subtilis translesion polymerase Pol Y1 is not strongly recruited to sites of replication upon different types of DNA damage

Dear Dr Thrall,

We are pleased to inform you that your manuscript entitled "The B. subtilis translesion polymerase Pol Y1 is not strongly recruited to sites of replication upon different types of DNA damage" has been formally accepted for publication in PLOS Genetics! Your manuscript is now with our production department and you will be notified of the publication date in due course.

The corresponding author will soon be receiving a typeset proof for review, to ensure errors have not been introduced during production. Please review the PDF proof of your manuscript carefully, as this is the last chance to correct any errors. Please note that major changes, or those which affect the scientific understanding of the work, will likely cause delays to the publication date of your manuscript.

Soon after your final files are uploaded, unless you have opted out or your manuscript is a front-matter piece, the early version of your manuscript will be published online. The date of the early version will be your article's publication date. The final article will be published to the same URL, and all versions of the paper will be accessible to readers.

For Research Articles, you will receive an invoice from PLOS for your publication fee after your manuscript has reached the completed accept phase. If you receive an email requesting payment before acceptance or for any other service, this may be a phishing scheme. Learn how to identify phishing emails and protect your accounts at https://explore.plos.org/phishing.

Thank you again for supporting PLOS Genetics and open-access publishing. We are looking forward to publishing your work!

With kind regards,

Janani Seenivasan

PLOS Genetics

On behalf of:

The PLOS Genetics Team

Carlyle House, Carlyle Road, Cambridge CB4 3DN | United Kingdom

plosgenetics@plos.org | +44 (0) 1223-442823

plosgenetics.org | Twitter: @PLOSGenetics
